# Education, biological ageing, all-cause and cause-specific mortality and morbidity: UK biobank cohort study

**DOI:** 10.1016/j.eclinm.2020.100658

**Published:** 2020-11-19

**Authors:** Marc Chadeau-Hyam, Barbara Bodinier, Roel Vermeulen, Maryam Karimi, Verena Zuber, Raphaële Castagné, Joshua Elliott, David Muller, Dusan Petrovic, Matthew Whitaker, Silvia Stringhini, Ioanna Tzoulaki, Mika Kivimäki, Paolo Vineis, Paul Elliott, Michelle Kelly-Irving, Cyrille Delpierre

**Affiliations:** a Department of Epidemiology and Biostatistics, School of Public Health, Imperial College London, London, United Kingdom; b MRC Centre for Environment and Health, Imperial College, London, United Kingdom; c Institute for Risk Assessment Sciences (IRAS), Utrecht University, Utrecht, Netherlands; d LEASP, UMR 1027, Inserm-Université Toulouse III Paul Sabatier, Toulouse, France; e University Centre for General Medicine and Public Health (UNISANTE), Lausanne University, Lausanne, Switzerland; f Unit of Population Epidemiology, Department of Primary Care, Geneva University Hospitals, Geneva, Switzerland; g Department of Hygiene and Epidemiology, University of Ioannina Medical School, Ioannina, Greece; h Department of Epidemiology and Public Health, University College London, London, United Kingdom; i Clinicum, Faculty of Medicine, University of Helsinki, Helsinki, Finland; j Italian Institute for Genomic Medicine IIGM, Torino, Italy; k National Institute for Health Research, Biomedical Research Centre, Imperial College London, London, United Kingdom; l Health Data Research UK London at Imperial College London, London, London, United Kingdom

**Keywords:** Biomarkers, Biological ageing, Social embedding, Allostatic load mortality, Incidentpathologies, Prospective cohort, Uk biobank, Mendelian randomisation

## Abstract

**Background:**

Socioeconomic position as measured by education may be embodied and affect the functioning of key physiological systems. Links between social disadvantage, its biological imprint, and cause-specific mortality and morbidity have not been investigated in large populations, and yet may point towards areas for public health interventions beyond targeting individual behaviours.

**Methods:**

Using data from 366,748 UK Biobank participants with 13 biomarker measurements, we calculated a Biological Health Score (BHS, ranging from 0 to 1) capturing the level of functioning of five physiological systems. Associations between BHS and incidence of cardiovascular disease (CVD) and cancer, and mortality from all, CVD, cancer, and external causes were examined. We explored the role of education in these associations. Mendelian randomisation using genetic evidence was used to triangulate these findings.

**Findings:**

An increase in BHS of 0.1 was associated with all-cause (HR = 1.14 [1.12–1.16] and 1.09 [1.07–1.12] in men and women respectively), cancer (HR = 1.11 [1.09–1.14] and 1.07 [1.04–1.10]) and CVD (HR = 1.25 [1.20–1.31] and 1.21 [1.11–1.31]) mortality, CVD incidence (HR = 1.15 [1.13–1.16] and 1.17 [1.15–1.19]). These associations survived adjustment for education, lifestyle-behaviours, body mass index (BMI), co-morbidities and medical treatments. Mendelian randomisation further supported the link between the BHS and CVD incidence (HR = 1.31 [1.21–1.42]). The BHS contributed to CVD incidence prediction (age-adjusted C-statistic = 0.58), other than through education and health behaviours.

**Interpretation:**

The BHS captures features of the embodiment of education, health behaviours, and more proximal unknown factors which all complementarily contribute to all-cause, cancer and CVD morbidity and premature death.

**Research in context**

**Evidence before this study**

The concept of social embodiment postulates that the human environment, through its physical, chemical, psychosocial stresses, solicits several adaptative processes, which over the life-course leave a sustained biological mark and can be measured using biomarkers. Measures of the wear and tear of the physiological systems involved in the stress response capture socially patterned features of biological ageing and have been found to relate to mortality and functional outcomes. However, there is limited evidence on the extent to which social factors, measures of its embodiment, socially patterned exposures and behaviours may contribute to these associations, and in particular to incident chronic diseases.

**Added value of this study**

We used a composite score to measure the physiological wear-and-tear of three key systems (inflammatory, metabolic and cardiovascular) and the functioning of two organs (kidney and liver) based on the measurement of 13 biomarkers in 366,748 UK Biobank participants who were free of cancer and CVD at enrolment. Descriptive analyses showed a strong education gradient in the score that was not fully explained by later-in-life socially patterned behaviours and exposures, which emphasises the ability of such composite scores to capture, in adulthood, features of social embodiment that could not be captured by established health risk factors.

We subsequently related our BHS to all-cause, cancer and CVD mortality and to cancer and CVD incidence. We found that the BHS was associated with increased all-cause, cancer and CVD mortality, and cancer and CVD incidence. Despite strong gradients in the BHS across education groups, these associations were only mildly attenuated upon adjustment for education, though larger attenuations were observed while adjusting for other factors, in particular BMI.

**Implications of all the available evidence**

Overall our results suggest that composite scores such as ours may act as markers of biological ageing, capturing features of social embodiment as well as biological effects of more proximal behaviours and health risk factors. Our study is the first to show a strong social gradient in a measure of biological wear and tear and we show that this role is independent of (i) education as a marker of social determinants of health, and (ii) established (and potentially socially patterned) health risk factors. We further show that biological age complements health behaviours to improve prediction of CVD incidence. Understanding the alternative mechanisms linking biological ageing markers, social determinants and adverse health outcomes, independently of behaviours, should be a research priority to identify novel targets for policy interventions.

Alt-text: Unlabelled box

## Introduction

Social inequalities have been described over time and between populations showing that social disadvantage was associated with poorer health and functional outcomes and earlier mortality [1,2]. While evidence has accumulated to relate social experiences, health behaviours and outcomes [3], [4], [5], [6], the way social environment over the life-course affects biological functioning is poorly understood. Once behavioural factors are accounted for, the social gradient in health remains pervasive, pointing to alternative mechanisms linking the social to the biological environments. This has been formalised in the concept of social embodiment, which postulates that the human environment, through its physical, chemical and psychosocial stresses, precipitates several adaptative processes [2]. In the life-course, these processes can be measured using biomarkers. Multi-system scores such as the allostatic load measure the physiological ‘wear-and-tear’ of key biological systems involved in the stress response across the life-course [7] and have been associated with subsequent functional decline and mortality [8,9]. These have appeared to be socially patterned and to capture features of biological ageing at different life-stages [10]. There is limited evidence to date on the extent to which social factors, measures of its embodiment, socially patterned exposures and behaviours may contribute to these associations and – in particular – to risk of incident chronic disease. Understanding the complex links between social disadvantage, biological health and chronic disease may highlight areas for public health action beyond focussing on individual-level behaviours.

As an extension of the allostatic load, we previously developed a Biological Health Score (BHS) which included physiological systems not directly related to the stress response [11], and showed that participants with lower educational attainment had higher BHS values (i.e. higher biological risk) independently of socially patterned exposures and behaviours. This suggested that composite scores are able to capture aspects of the effect of social environment on biological functioning [11].

In the present study, using data from UK Biobank participants, we investigate the link between education, biological functioning (as measured by the BHS), and all-cause and cause-specific mortality, as well as cardiovascular disease (CVD) and cancer incidence. We calculate baseline BHS as a proxy for biological ageing and (i) evaluate the association between the BHS and prospective health outcomes, (ii) investigate if and to what extent these links are explained by education and socially patterned exposures, and (iii) use genetic evidence to further triangulate the association between the BHS and health outcomes through Mendelian randomisation.

## Methods

### Study population

The UK Biobank study includes 502,536 volunteers from the UK aged 37–73 years at entry between 2006 and 2010. Participants were recruited throughout England, Wales and Scotland and included socioeconomic and ethnic heterogeneity and an urban-rural mix of the population [12]. Each participant completed an electronic informed consent form and filled a computer-based questionnaire on life-course exposures, medical history and treatments. Participants underwent clinical measurements using standardised protocols in the 22 assessment centres. They donated a blood sample for long-term storage from which (i) DNA was extracted for genotyping[13,14], and (ii) a set of 30 biomarkers were measured [15].

### Socioeconomic position and covariates

As a more extensive measure of socioeconomic position capturing both the length of education (through the highest diploma achieved), but also features of future occupation of the participant, we used educational attainment as a proxy for early-life socio-economic position. It was coded in three categories: high (college or university degree), intermediate (A/AS levels or equivalent, O levels/GCSEs or equivalent), and low (none of the aforementioned).

We considered as potential confounders the following variables previously used when analysing the BHS [11]:

(i) the number of comorbidities (recoded into two categories: none, one or more comorbidities) among conditions that may potentially affect at least one physiological system of interest (supplementary methods);
(ii) the number of pharmacological treatments, which were recoded into three categories: none, one, and two or more reported treatments;
(iii) smoking and physical activity, which were recoded into binary variables indicating if the participant had ever smoked or was undertaking vigorous physical activity for more than 10 min, at least once a week;
(iv) alcohol consumption coded into 4 categories: Non-drinker (Never); Social drinker (One to three times a month, Special occasions only); Moderate drinker (Once or twice a week); Daily drinker (more than three times a week);
(v) Body mass index (BMI, kg/m^2^) grouped using the following four categories: <25, 25–29.99, 30–39.99, and ≥40.

### Biomarkers, BHS calculation, and genotypes

Similarly to the allostatic load, the BHS includes measures of the physiological wear and tear of the inflammatory, cardiovascular, and metabolic systems, and is further complemented by markers of the level of functioning of two key organs: liver and kidney [11]. As detailed in Box 1, of the 30 biomarkers available in the UK Biobank dataset, we selected those related to any of the aforementioned physiological systems (*N* = 13). The BHS was calculated by first dichotomising the distribution of each biomarker, considering an individual being ‘at risk’ for that specific biomarker if the measured level of that biomarker was in the extreme age and gender-specific quartile. As a conservative assumption, if the value of a given biomarker was missing, a null sub-score was allocated to the individual for that specific biomarker. The per-system sub-score was obtained by summing biomarker-specific scores across all biomarkers within the system, and the BHS by summing each system-specific sub-score. Scores were scaled to ensure that the BHS and each system-specific sub-score were measured on the same scale and not driven by the number of biomarkers they included.

DNA samples from UK Biobank participants were genotyped using custom Affymetrix arrays. After pre-processing, quality-control filtering of both samples and genetic variants [16], the genetic data included *N* = 672,345 SNPs assayed in 488,377 participants.

### Health outcomes

As the two main causes of deaths in the UK, we focused on cancer and CVD [17], and, as a negative control group not reported to be associated with the level of functioning of any of the physiological systems included in our score, we also investigated external causes of death including suicide and accidents (ICD-10: V00-V99, W00-W99, X00-X99, Y00-Y99). The cause of death of the UK Biobank participants was obtained by linkage to the national death registers. The health status of the UK Biobank participants was followed up (range 0.27 to 12.04 years) by linkage to NHS central registers: incident cases of cancer were identified through linkage to cancer and hospital registers and incident CVD cases, using hospital registers and nurse-administered questionnaires. As previously reported [2], we adopted a broad definition of cancer, including all sites (see Supplementary Table 1, ICD10 codes: C00-C97 and D00-D48). For CVD, we adopted a disease definition including coronary artery disease (CAD), angina, stroke and related outcomes [18] (Supplementary Table 2, ICD10 codes: G45, I20-I25, I63, I64, I67.2, I67.9, and OPCS-4 code K).

### Data analysis

#### Participant selection

Of the 502,536 participants, we excluded (i) 381 participants with inconsistent genetic and reported sex or inconsistent date of death, (ii) a total of 105,950 (*N* = 77,772 cancer, *N* = 34,230 CVD) prevalent cases, (iii) 2138 individuals with all biomarkers missing, (iv) 26,166 participants with missing information on education or one or more missing covariate, and (vi) one participant with uncertain cancer status (Supplementary Figure 1), leaving 366,748 (171,193 men, 195,555 women) participants free of cancer and CVD at baseline. Age at recruitment was grouped into the following three categories: <50, 50–64, and >64 years old, giving similar number of participants in each age group among men and women (Supplementary Table 3).

We calculated the BHS (and system-specific sub-scores) for each participant and explored age and education gradients in the scores using linear models (Supplementary Methods). Up to March 1st, 2016 (i) a total of 14,396 deaths were observed, including 8,014, 1,899, and 488 from cancer, CVD, and external causes, respectively (Supplementary Table 4-A), and (ii) 52,443 and 15,653 cases of cancer and CVD were diagnosed (Supplementary Table 4-B).

#### Survival analyses

Univariate and multivariate Cox proportional hazard regression models using age as timescale were fitted to estimate hazard ratios (HRs) for the BHS on health outcomes. We set the BHS (or system-specific sub-score) as predictor, and sequentially adjusted for education level, lifestyle behaviours (smoking, physical activity, and alcohol consumption), BMI, and co-morbidities and treatments. Analyses were conducted in men and women separately for (i) all-cause, (ii) cancer, (iii) CVD, and (iv) external-cause mortality, and (v) cancer and (vi) CVD incidence (Supplementary Methods). Effect size estimates were expressed as hazard ratios per 0.1 increase in the BHS (or system-specific sub-scores). We adopted the same sequential adjustment procedure for models setting education as predictor, which were further adjusted for behaviours, BMI, co-morbidities and treatments, and the BHS.

To assess the predictive performances of the models investigated, we used the same Cox regression models using age as timescale to derive the distribution of the age-adjusted 10-year event-free survival probabilities in cases and non-cases, as well as age-adjusted Harrell's C-statistic. C-statistics were also calculated for survival models unadjusted for age, by taking time elapsed since enrolment as timescale and additionally including chronological age as a predictor.

The validity of the proportional hazard assumption was evaluated by visual inspection of the Schoenfeld residuals.

#### Sensitivity analysis

To check the potential role of ethnicity, we ran our survival models adjusting for ethnicity. Ethnicity was self-reported in the UK Biobank and reflects social aspects of minority communities.

As an unsupervised alternative to the BHS, we constructed a continuous biomarker score based on principal component analysis[19,20] to summarise the information in the biomarker data and chose the first component of (i) the 13 biomarkers as an alternative to the BHS and (ii) biomarkers included in each system, as an alternative to each system-specific score in our survival analyses.

#### Mendelian randomisation

We adopted a one-sample two-stage least squares Mendelian randomisation (MR) approach [21] relying first on the identification of genetic instruments which were used to estimate the instrumentally explained (exposure-independent) part of the BHS. The selection of genetic variants as instrumental variables was done using a linear model coupled with a stringent pruning strategy (Supplementary Methods) [22]. We performed series of sensitivity analyses for the selection of instrumental variables using (i) the BOLT-LMM model, as an alternative with relaxed normality assumptions, (ii) using different values of the significance level to select variants in the clumping step, and (iii) using different values of r^2^ for the pruning of SNPs in high LD. In the second stage, the genetically predicted BHS was regressed against the health outcomes and resulting effect size estimates were used to infer the causal effect of the BHS on each outcome (Supplementary methods). The second stage regression was based on a proportional hazard Cox model [23] adjusted for sex, and the first 10 principal components capturing the latent population structure. The univariable MR model including only BHS as risk factor was extended to a multivariable Mendelian Randomisation (MVMR) [24], where the survival model also included the instrumental estimate of the education variable as additional risk factor, using the same genetic instruments in order to account and adjust for a pleiotropic effect via education.

All statistical analyses were performed using R v3•6•1 in the RStudio environment and PLINK v1.90p 64-bit (16 Apr 2016).

### Ethical approval

Ethical approval for the nurse visit was obtained from the National Research Ethics Service (Reference: 10/H0604/2). Participants gave written consent for blood sampling (McFall et al. 2014).

### Role of the funding source

The funders had no role in the design and conduct of the study; collection, management, analysis, and interpretation of the data; and preparation, review, or approval of this manuscript.

## Results

### Data and BHS exploration

Characteristics of the study participants are given in Table 1 for each age group in men and women separately. Mean follow-up for all-cause and cause-specific mortality were comparable (mean person-years ranging from 4.24 to 5.00). Shorter follow-up was observed for cancer and CVD incidence (from 3.94 to 4.22 person-years).

**Table 1:** Description of the study population across all covariates by gender and age class. The percentages given in parenthesis are the proportion of individuals relative to the total number of individuals in that age class and gender. The average and standard errors of person-years to death, the first event (cancer or CVD incidence) or end of follow-up (for living, cancer and CVD free individuals at the end of follow-up) are also reported for men and women separately.

	Women (%)			Men (%)		
Age Group	<50 (*N* = 51,726)	50–64 (*N* = 113,793)	>64 (*N* = 30,036)	<50 (*N* = 46,127)	50–64 (*N* = 96,930)	>64 (*N* = 28,136)
Education						
Low	1716 (3.32%)	17,078 (15.01%)	8938 (29.76%)	2494 (5.41%)	13,952 (14.39%)	7799 (27.72%)
Intermediate	28,433 (54.97%)	58,888 (51.75%)	14,344 (47.76%)	24,964 (54.12%)	46,695 (48.17%)	12,367 (43.95%)
High	21,577 (41.71%)	37,827 (33.24%)	6754 (22.49%)	18,669 (40.47%)	36,283 (37.43%)	7970 (28.33%)
Co-morbidities						
None	41,301 (79.85%)	93,630 (82.28%)	25,557 (85.09%)	38,759 (84.03%)	84,572 (87.25%)	24,765 (88.02%)
One or more	10,425 (20.15%)	20,163 (17.72%)	4479 (14.91%)	7368 (15.97%)	12,358 (12.75%)	3371 (11.98%)
Number of treatments						
None	29,786 (57.58%)	52,826 (46.42%)	9937 (33.08%)	32,096 (69.58%)	53,296 (54.98%)	10,890 (38.7%)
One	9245 (17.87%)	19,137 (16.82%)	4991 (16.62%)	6501 (14.09%)	14,726 (15.19%)	4484 (15.94%)
Two or more	12,695 (24.54%)	41,830 (36.76%)	15,108 (50.3%)	7530 (16.32%)	28,908 (29.82%)	12,762 (45.36%)
Smoking						
Never	33,160 (64.11%)	68,118 (59.86%)	17,891 (59.57%)	27,408 (59.42%)	48,812 (50.36%)	11,802 (41.95%)
Yes	18,566 (35.89%)	45,675 (40.14%)	12,145 (40.43%)	18,719 (40.58%)	48,118 (49.64%)	16,334 (58.05%)
Sports activity						
At least one sport	33,486 (64.74%)	67,856 (59.63%)	17,364 (57.81%)	34,170 (74.08%)	64,110 (66.14%)	17,702 (62.92%)
None	18,240 (35.26%)	45,937 (40.37%)	12,672 (42.19%)	11,957 (25.92%)	32,820 (33.86%)	10,434 (37.08%)
Alcohol consumption						
Non-drinker	3888 (7.52%)	9244 (8.12%)	3320 (11.05%)	2953 (6.4%)	4893 (5.05%)	1561 (5.55%)
Social drinker	14,767 (28.55%)	29,904 (26.28%)	8605 (28.65%)	8755 (18.98%)	13,864 (14.3%)	4044 (14.37%)
Moderate drinker	14,795 (28.6%)	29,221 (25.68%)	6990 (23.27%)	13,867 (30.06%)	24,080 (24.84%)	6606 (23.48%)
Daily drinker	18,276 (35.33%)	45,424 (39.92%)	11,121 (37.03%)	20,552 (44.56%)	54,093 (55.81%)	15,925 (56.6%)
Body Mass index (Kg/m^2^)						
Below 25	24,934 (48.2%)	45,471 (39.96%)	10,762 (35.83%)	13,098 (28.4%)	24,870 (25.66%)	7206 (25.61%)
Above 25 and below 30	16,470 (31.84%)	42,411 (37.27%)	12,537 (41.74%)	22,548 (48.88%)	48,170 (49.7%)	14,686 (52.2%)
Above 30 and below 40	9051 (17.5%)	23,371 (20.54%)	6295 (20.96%)	9899 (21.46%)	22,664 (23.38%)	6039 (21.46%)
above 40	1271 (2.46%)	2540 (2.23%)	442 (1.47%)	582 (1.26%)	1226 (1.26%)	205 (0.73%)
Person-years: mean (s.d.)						
All-cause deaths		4.94 (1.95)			4.87 (2.00)	
Cancer deaths		5.00 (1.89)			4.98 (1.95)	
CVD deaths		4.61 (2.24)			4.55 (2.08)	
External cause deaths		4.24 (2.00)			4.37 (2.04)	
Other cause deaths		4.97 (1.99)			4.92 (2.03)	
Alive at the end of follow-up		7.58 (0.88)			7.58 (0.88)	
Cancer incident cases		3.94 (2.18)			4.07 (2.17)	
CVD incident cases		4.22 (2.18)			4.21 (2.15)	
Cancer and CVD-free individuals		7.57 (0.88)			7.57 (0.88)	

**Fig. 1:**
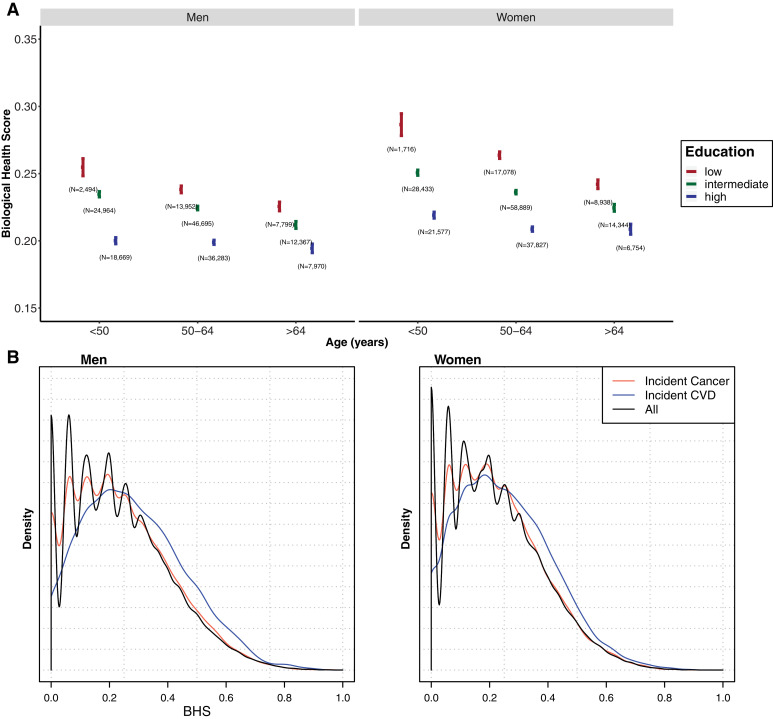
(A) Distribution of the BHS by age groups and education levels for the study population excluding prevalent cases. For each category the point estimate of the mean BHS is represented by a bullet and the vertical line represents the 2.5–97.5% confidence interval of the score in that category for men (left) and women (right). Low, intermediate and high education are represented in red, green and blue, respectively. For both genders and within each age class, differences in mean BHS by education category were statistically significant (*p* < 0.001, mean BHS in the high education category as reference) as were trends in BHS across the three education categories for both genders and within each age class (*p* < 0.001). (B) BHS distribution for incident cancer (red), and CVD (blue) cases and for full population at the latest follow-up (black) in men (left), and women (right).

We observe a social gradient in BHS across education groups in both men and women with higher scores (i.e. higher biological risk) in the lower education group (Fig. 1-A), and slightly higher BHS values in individuals diagnosed with CVD during the follow-up compared with non-cases (Fig. 1-B). We found differences in the BHS according to all covariates (Supplementary Table 5, *p* < $10^{-16}$). Similarly, we found, in both genders and all age groups, a downward gradient leading to higher metabolic, cardiovascular, inflammatory, and liver scores in the lowest education group (Supplementary Table 6). The effect of education on the BHS was stronger in younger age groups, and, irrespective of age, an attenuation of that effect was observed while adjusting for each covariate, in particular BMI (Supplementary Table 7).

### Survival models: mortality and incidence

Univariate Cox models using the scores as predictor indicate that the BHS and all system-specific sub-scores were associated with higher all-cause, cancer and CVD mortality. Estimated HR (mean [2.5^th^–97.5^th^ percentiles]) for 0.1 higher BHS were 1.14 [1.12–1.16], 1.11 [1.09–1.14], 1.25 [1.20–1.31] respectively in men (Table 2-A) and 1.09 [1.07–1.12], 1.07 [1.04–1.10], 1.21 [1.11–1.31] in women (Table 2-B). The metabolic, inflammatory and liver scores were all associated with all-cause, cancer and CVD mortality, in men and women.

**Table 2:** Hazard Ratios (HR) (and 95% confidence interval) from the univariate Cox model including the BHS or each system-specific sub-score as predictor. HRs were expressed for a 0.1 increase in the score, and models were run for all-cause, cancer, CVD and external-cause mortality and for cancer and CVD incidence in men (A) and women (B), separately.

	Mortality			Incidence		
	All-cause (*N* = 7144 deaths)	Cancer (*N* = 3914 deaths)	CVD (*N* = 846 deaths)	External cause (*N* = 315 deaths)	Cancer (*N* = 43,772 cases)	CVD (*N* = 11,653 cases)
	HR [95% CI] p-value	HR [95% CI] p-value	HR [95% CI] p-value	HR [95% CI] p-value	HR [95% CI] p-value	HR [95% CI] p-value
**A. Men**	*N* = 4428	*N* = 2225	*N* = 681	*N* = 220	*N* = 20,962	*N* = 7925
BHS	1.14 [1.12–1.16] 7.63 × 10^−44^	1.11 [1.09–1.14] 1.00 × 10^−16^	1.25 [1.20–1.31] 2.70 × 10^−24^	0.99 [0.91–1.08] 8.49 × 10^−01^	1.02 [1.01–1.03] 1.01 × 10^−04^	1.15 [1.13–1.16] 1.28 × 10^−93^
System-specific sub-score						
*Metabolic*	1.05 [1.04–1.06] 2.66 × 10^−^14	1.04 [1.02–1.06] 1.27 × 10^−05^	1.14 [1.11–1.18] 5.80 × 10^−20^	0.97 [0.92–1.03] 3.45 × 10^−01^	1.01 [1.00–1.01] 4.20 × 10^−03^	1.12 [1.11–1.13] 6.43 × 10^−138^
*Cardiovascular*	1.05 [1.04–1.06] 2.99 × 10^−26^	1.04 [1.02–1.05] 1.32 × 10^−07^	1.10 [1.07–1.12] 1.06 × 10^−15^	1.04 [1.00–1.08] 6.13 × 10^−02^	1.00 [1.00–1.01] 2.22 × 10^−01^	1.05 [1.05–1.06] 1.31 × 10^−51^
*Inflammatory*	1.07 [1.06–1.08] 3.20 × 10^−57^	1.06 [1.05–1.08] 7.09 × 10^−22^	1.09 [1.07–1.11] 3.51 × 10^−15^	1.01 [0.97–1.05] 7.00 × 10^−01^	1.01 [1.01–1.02] 1.20 × 10^−06^	1.04 [1.03–1.05] 6.72 × 10^−32^
*Liver*	1.03 [1.02–1.04] 1.89 × 10^−11^	1.03 [1.01–1.04] 2.06 × 10^−04^	1.04 [1.02–1.07] 3.61 × 10^−04^	1.02 [0.98–1.06] 3.73 × 10^−01^	1.00 [1.00–1.01] 1.63 × 10^−01^	1.02 [1.02–1.03] 1.42 × 10^−10^
*Kidney*	0.99 [0.98–0.99] 1.24 × 10^−03^	1.00 [0.98–1.01] 4.20 × 10^−01^	0.99 [0.97–1.01] 5.75 × 10^−01^	0.95 [0.91–0.99] 2.05 × 10^−02^	1.00 [1.00–1.00] 8.60 × 10^−01^	1.00 [0.99–1.01] 8.59 × 10^−01^
**B. Women**	*N* = 2716	*N* = 1689	*N* = 165	*N* = 95	*N* = 22,810	*N* = 3728
BHS	1.09 [1.07–1.12] 8.38 × 10^−16^	1.07 [1.04–1.10] 8.54 × 10^−06^	1.21 [1.11–1.31] 1.21 × 10^−05^	0.94 [0.83–1.07] 3.51 × 10^−01^	1.02 [1.01–1.03] 1.07 × 10^−05^	1.17 [1.15–1.19] 6.84 × 10–65
System-specific sub-score						
*Metabolic*	1.04 [1.03–1.06] 3.91 × 10–08	1.03 [1.01–1.05] 4.44 × 10^−04^	1.18 [1.12–1.24] 9.45 × 10^−10^	0.94 [0.86–1.02] 1.54 × 10^−01^	1.01 [1.01–1.02] 9.69 × 10^−06^	1.12 [1.11–1.14] 3.56 × 10–87
*Cardiovascular*	1.03 [1.02–1.04] 1.29 × 10^−06^	1.01 [1.00–1.03] 8.94 × 10^−02^	1.08 [1.03–1.13] 1.47 × 10^−03^	1.00 [0.94–1.07] 9.96 × 10^−01^	1.01 [1.01–1.01] 7.99 × 10^−06^	1.05 [1.04–1.06] 6.72 × 10^−26^
*Inflammatory*	1.04 [1.03–1.05] 2.81 × 10–12	1.03 [1.02–1.05] 2.49 × 10^−05^	1.07 [1.02–1.12] 4.15 × 10^−03^	0.99 [0.92–1.06] 7.05 × 10^−01^	1.00 [1.00–1.01] 3.59 × 10^−02^	1.05 [1.04–1.06] 1.15 × 10–19
*Liver*	1.03 [1.02–1.04] 1.51 × 10^−07^	1.02 [1.01–1.04] 3.06 × 10^−03^	1.04 [0.99–1.09] 8.38 × 10^−02^	0.99 [0.93–1.06] 7.49 × 10^−01^	1.00 [1.00–1.01] 8.45 × 10^−02^	1.04 [1.03–1.05] 5.31 × 10^−15^
*Kidney*	1.00 [0.99–1.01] 9.93 × 10^−01^	1.00 [0.99–1.01] 9.05 × 10^−01^	0.98 [0.95–1.02] 4.20 × 10^−01^	0.99 [0.94–1.04] 6.23 × 10^−01^	1.00 [1.00–1.00] 9.83 × 10^−01^	1.01 [1.00–1.01] 1.16 × 10^−01^

Results also suggested that the BHS was associated with a small increase in cancer incidence (mean HR [2.5^th^–97.5^th^ percentiles]: 1.02 [1.01–1.03] and 1.02 [1.01–1.03] in men and women, respectively). Much stronger effects were estimated for CVD incidence, mean HR [2.5th97.5th] percentiles were 1.15 [1.13–1.16] and 1.17 [1.15–1.19] in men and women respectively (Table 2). All system-specific sub-score (except kidney score) were associated with CVD incidence and (weaker) associations with cancer incidence of the metabolic, cardiovascular (in women only) and the inflammatory sub-scores were observed.

The effect of the BHS – or its continuous alternative, the score of the first principal component summarising the 13 biomarkers – on all-cause, cancer and CVD mortality, and cancer and CVD incidence was similar or mostly greater than that of any of the individual biomarker (Supplementary Figure 2).

### Attenuation analyses

**Fig. 2:**
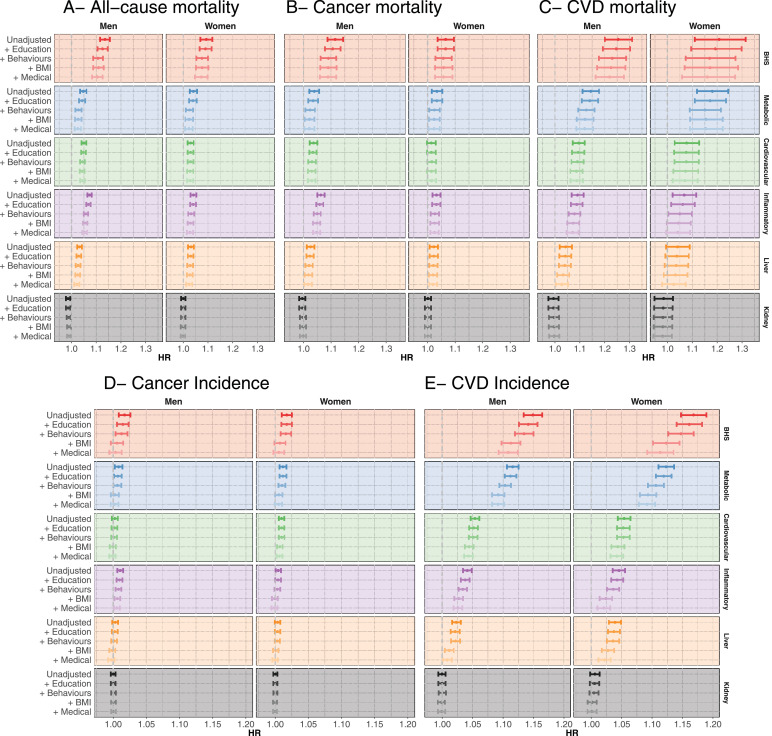
Hazard ratio from the proportional hazard Cox model relating (A) all cause, (B) cancer and (C) cardiovascular mortality, cancer (D), and CVD (E) incidence and the Biological Health Score (BHS, red), the metabolic (blue), the cardiovascular (green), the inflammatory (purple), the kidney (orange), and the liver (grey) sub-scores. Hazard ratios are expressed as a risk change per 0.1 increase in the score. Results are presented for men (left) and women (right) and for the unadjusted model, for models sequentially adjusted for education group, lifestyle behaviours (smoking, physical activity, and alcohol consumption), BMI and medical status (number of comorbidities and treatments).

Multivariate Cox models showed modest attenuation of the effect of the BHS and all system-specific sub-scores on all cause, cancer and CVD mortality when adjusting for education. Stronger effect attenuation was observed when adjusting for lifestyle behaviours (Fig. 2A-C, and Supplementary Tables 8–10). In the fully adjusted models, the HR remained statistically significant for the BHS on all-cause, cancer and CVD mortality, as was also the case for the metabolic, cardiovascular (except for cancer mortality in women), and inflammatory (except for CVD mortality in women) sub-scores. The BHS or system-specific sub-scores were not associated with external causes of mortality (*N* = 315 deaths), irrespective of the covariates included in the model (Supplementary Figure 3 and Supplementary Table 11).

Analyses of incident diseases showed, for both cancer and CVD incidence, a limited attenuation of the effect of the BHS and system-specific sub-scores when adjusting for education (Fig. 2DE, Supplementary Tables 12–13). Stronger effect attenuations were observed when BMI was included in the model, particularly for CVD incidence. None of the BHS and system-specific sub-scores were associated with cancer incidence in the fully adjusted models. In contrast, and especially amongst women, we observed stronger effects of BHS on CVD incidence. Fully adjusted HRs were 1.11 [1.09–1.12], and 1.11 [1.09–1.14] in men and women respectively, and were greater than effect size estimates of each system-specific sub-score, including metabolic (HR 1.09 [1.08–1.10] and 1.09 [1.08–1.11]), cardiovascular (HR 1.04 [1.04–1.05] and 1.04 [1.03–1.05]), and inflammatory scores (1.03 [1.02–1.03] and 1.02 [1.01–1.03]).

Further adjusting our survival models for ethnicity (Supplementary Figure 4) identified exactly the same associations and did not change the effect size estimates.

Sensitivity analyses considering principal components scores instead of our composite scores did not affect our conclusions (Supplementary Figures 5–6 for mortality and incidence, respectively).

Schoenfeld residuals plots (Supplementary Figures 7–8 for mortality and incidence analyses, respectively) did not indicate violation of the proportionality assumption.

### Education and socially patterned exposures

**Fig. 3:**
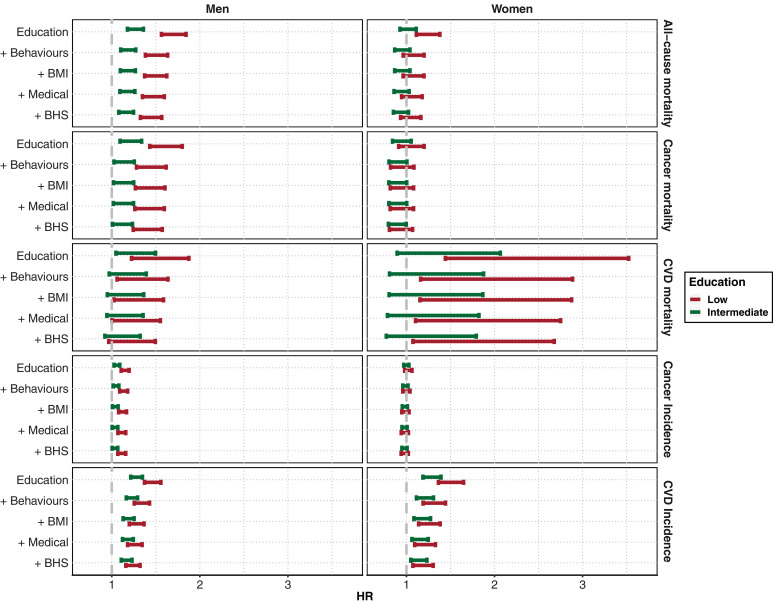
Hazard ratio from the proportional hazard Cox model relating all-cause, cancer, and cardiovascular mortality, cancer and cardiovascular disease incidence, and education level (considering the high education group as reference). Results are presented for men (left) and women (right) and for the unadjusted model, and for models sequentially adjusted for behaviours and lifestyle (smoking, physical activity, and alcohol consumption), BMI, comorbidities and treatments, and BHS.

Survival models including education as the independent variable showed, in both men and women and across all five health outcomes, (i) higher HRs for the low education group, and (ii) a stronger effect in men for all health outcomes except CVD incidence (Fig. 3). Strong effect attenuation was observed, irrespective of gender and disease outcome, in the model including lifestyle behaviours, while the inclusion of the BHS in the model yielded a limited attenuation of the HRs. In the fully adjusted model, education was significantly associated with CVD incidence in both genders and all education groups (HR = 1.17 [1.11–1.23]; 1.14 [1.05–1.23] for intermediate education in men and women, respectively; 1.24 [1.16–1.32]; 1.18 [1.07–1.30] for low education in men and women respectively), in men only with all-cause mortality (HR = 1.44 [1.32–1.57], and 1.16 [1.08–1.25] for low and intermediate education) and cancer mortality (HR = 1.40 [1.24–1.57], and 1.12 [1.01–1.23] for low and intermediate education), in men only for the lower education group with cancer incidence (HR = 1.11 [1.07–1.16]), and in women only for the lower education group with CVD mortality (1.70 [1.07–2.68]).

### Mendelian randomisation

The GWAS of the BHS identified 172 genetic variants within autosomes which we used as instrumental variables in a one-sample MR analysis (Supplementary Table 14). These explained 2.0% of the variance of the BHS. Genetically predicted BHS (Table 3) was significantly associated with CVD incidence (Base model, HR = 1.31, *p*-value < $10^{-10}$) but not with other outcomes. In order to account for a potential pleiotropic pathway via education we performed MVMR adjusting for genetically predicted education, where the effect of the BHS was not attenuated (HR = 1.30, *p*-value < $10^{-9}$). This suggests that the link between the BHS and CVD incidence is independent of education. Sensitivity analyses with different parameters for the selection of instrumental variables yielded highly consistent results (Supplementary Table 15).

**Table 3:** Results from the two-step least squares Mendelian randomisation approach. Causal effects (b) were estimated using a proportional hazard Cox model from a regression of the instrumentally explained BHS against all-cause, cancer and CVD mortality, and cancer and CVD incidence. Hazard Ratios (HRs) are expressed for a 0.1 increase in the score, and we report the p-value assessing if the causal effect is different from 0. We present estimates for the model adjusted for age, sex and the first 10 principal components capturing the latent structure of the UK Biobank population (Base model), and for the model additionally adjusted for education. Results from the multivariable Mendelian randomisation were adjusted for age, sex and the 10 first principal components capturing the latent structure of the UK Biobank population.

	Base model			Base model + Education			Base model + $\hat{\text{Education}}$		
	β	HR	p-value	β	HR	p-value	β	HR	p-value
All-cause mortality	0.03	1.03	6.09 × 10^−01^	0.00	1.00	9.39 × 10^−01^	0.02	1.02	7.33 × 10^−01^
Cancer mortality	−0.01	0.99	8.91 × 10^−01^	−0.04	0.96	5.61 × 10^−01^	−0.02	0.98	8.14 × 10^−01^
CVD mortality	0.12	1.12	4.43 × 10^−01^	0.10	1.11	5.16 × 10^−01^	0.11	1.11	4.82 × 10^−01^
Cancer incidence	0.01	1.01	6.29 × 10^−01^	0.01	1.01	7.33 × 10^−01^	0.01	1.01	6.68 × 10^−01^
CVD incidence	0.27	1.31	3.32 × 10^−11^	0.26	1.30	3.18 × 10^−10^	0.27	1.30	1.23 × 10^−10^

### Education, BHS, health behaviours and CVD incidence prediction

**Fig. 4:**
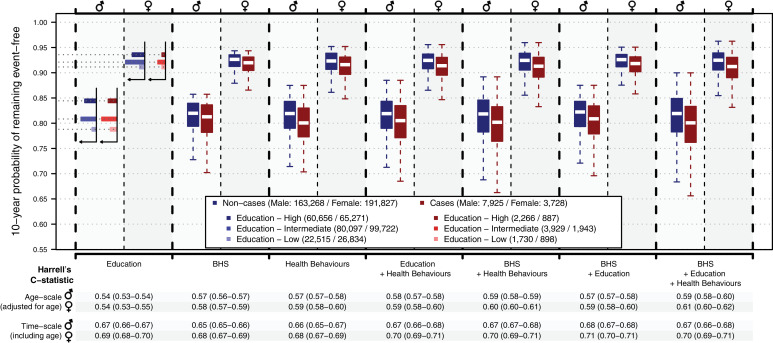
Distribution of individual probabilities from Cox proportional hazards using (i) Education, (ii) the BHS, (iii) Health behaviours (BMI, smoking, alcohol consumption, and physical activity), (iv) Education and Health behaviours, (v) BHS and Health behaviours, (vi) BHS and Education, and (vii) BHS, Education and Health behaviours as predictors of CVD incidence. Models were all adjusted for reported medical status (number of co-morbidities and medical treatments). Results are presented for non-cases (in blue) and cases (in red). In the model only including Education as predictor, the survival probability is discrete and has one value for each education group. The corresponding distribution is represented as a horizontal histogram showing the survival probability for the low (lightest tint), intermediate (medium tint), and high (solid colour) education group. Predictive performances of the models are summarised by their mean (and 2.5^th^ 97.5^th^ percentile confidence interval) of the Harrell's C-statistic for the age adjusted survival model (using age as timescale). We also report the Harrell's C-statistic for the model using time since enrolment as timescale and include age as predictor in all models. Results are presented for each model (X axis) in men and women separately.

We found that education was modestly predictive of CVD incidence (Fig. 4*C* = 0.54), and both BHS (*C* = 0.58) and health behaviours (*C* = 0.59) showed stronger predictive performances. Performances only marginally improved by adding education into the model including BHS and behaviours (*C* = 0.59 for both models). Combining the BHS and behaviours in the models yielded a C-statistic of 0.60, and the model including the BHS, behaviours, and education a C-statistic of 0.61. Unadjusted models including age as predictor showed stronger performances (C-statistics > 0.68) and were indicative of a marginal effect of the BHS over chronological age that was independent of education and health behaviours.

**Box 1:**
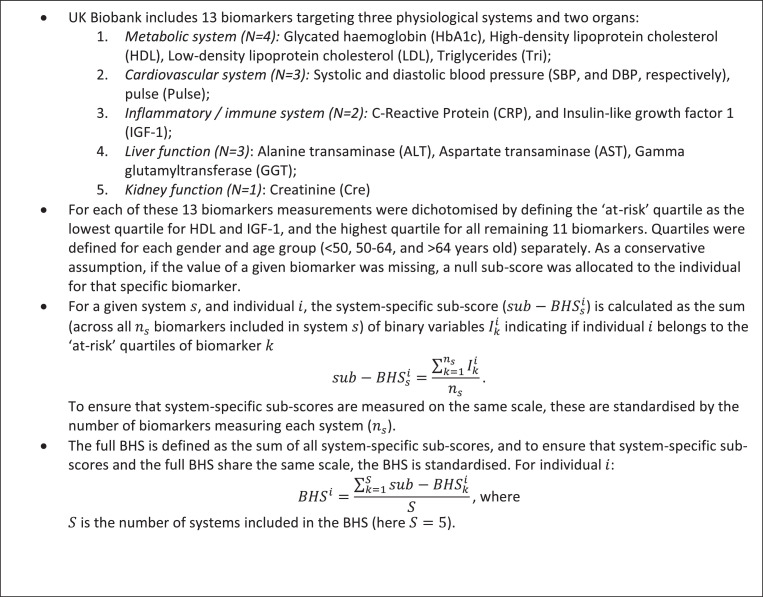
UK-Biobank biomarkers and BHS Calculation.

## Discussion

The UK Biobank cohort is one of few datasets combining information on time-resolved social factors and exposures, multiple biomarkers measurements, and reliable health outcomes and therefore offers unique opportunities to investigate the link between (i) social factors, (ii) biology, and (iii) chronic disease mortality and incidence. Our Biological Health Score (BHS) used measurements of 13 biomarkers in 366,748 UK-Biobank participants who were free of cancer and CVD at enrolment.

Descriptive analyses showed a strong education gradient in the BHS that was not fully explained by later-in-life socially patterned behaviours and exposures, which emphasises the ability of such composites score to capture, in adulthood, features of social embodiment that could not be captured by established health risk factors.

Our survival analyses were supportive of an association between the BHS and all system-specific sub-scores, except the kidney score, on all-cause, cancer and CVD mortality. The BHS and system-specific sub-scores were associated with a small increase in cancer incidence and a much larger increase in CVD incidence.

These findings are consistent with results of previous studies that linked markers of inflammation [25] or broader physiological scores[26], [27], [28], [29], [30], [31] and all-cause mortality. Unlike some other studies [32], our work was statistically powered to identify effects of markers of biological ageing and cause-specific mortality. Several studies have established a link between physiological markers and incidence of ischaemic heart disease [33], depressive symptoms [34], functional decline [8], and type-2 diabetes [35]. In the present study we were able, for the first time, to investigate the association between BHS and cancer and CVD incidence in the same population, and our results are supportive of a possible causal association with CVD incidence.

We identified a strong social gradient in the scores leading to higher biological risk in individuals with a lower education, which could not be fully explained by (socially patterned) behaviours and exposures [11]. Our analyses suggest that the effects of the scores on the incidence of cardiovascular disease and cancer and mortality from all, cardiovascular and cancer causes were only moderately affected by the inclusion of education in the model. In comparison, there was greater attenuation from the inclusion of lifestyle behaviours in the analyses of mortality and of BMI for disease incidence. In contrast to education, these factors are proximal to the measurement of the biochemical markers that make up the BHS, suggesting that proximal rather than earlier life factors play a more important role in the associations between the BHS score and biological risk.

We also identified an adverse effect of lower education on all five health outcomes consistent with previous studies. These were all strongly attenuated upon adjustment for lifestyle behaviours and to a lesser extent by the BHS.

Taken together, our results first suggest that the BHS has a stronger effect than each of its constituent biomarkers, and is able to capture features of the social embodiment that are independent of later-in-life health risk factors, as well as biological effects of more proximal behaviours and health risk factors. As such, our study emphasizes its ability to measure biological ageing. We find that education and the BHS capture different (largely independent) facets of socially patterned behaviours and biological information associated with disease risk. Our Mendelian randomisation results were indicative of a potential causal link between the BHS and CVD incidence, independent of education.

We found that the BHS captured disease-relevant features of biological ageing that were not related to chronological age. Age-adjusted predictive performances of the BHS in predicting CVD incidence were comparable to those of health behaviours and of a polygenic risk score recently developed and tested in the same study population [36]. We found that combining health behaviours, the BHS and, to a lesser extent, education, improves CVD incidence prediction, therefore supporting their complementarity.

Our study benefited from the size and quality of UK Biobank, which includes hundreds of thousands of individuals, followed-up and validated clinical data from registries enabling the investigation of cause-specific mortality (up to thousands of deaths) and incidence (up to tens of thousands of incident cases). Data also include biomarkers capturing features of biological ageing and genotype data enabling causal assessment through instrumental modelling. We selected participants who were free of cancer and CVD at enrolment, limiting the risk of reverse causality. Moreover, UK biobank includes genetic information on the participants, which allowed us to triangulate the evidence using a one-sample MR analysis.

The UK Biobank dataset has been reported to suffer from a healthy-volunteer selection bias [37], which hampers the generalisability of our findings: we may have over- or under-estimated the true effect sizes. In addition, previous studies have shown that the UK Biobank study population over-represented participants of white background. The resulting small number of observations in non-white participants precluded us from running stratified analyses on each ethnic group separately. The models adjusted for ethnicity showed very limited attenuation of effect size estimates. However, these should be considered with caution due to under-representation of ethnic minorities in our study population.

We considered a broad definition for cancer across multiple sites. The clinical heterogeneity across cancer types and sites may, together with the lower cancer incidence in the UK Biobank study compared to the general population [37], help explain why our results are weaker for cancer outcomes than for CVD.

Our focus on education as a proxy-measure for socio-economic position allowed us to examine in detail the association between education and health through the BHS. However, education captures only part of the complex social and structural factors that may be embodied over the life course. Future analyses using other measures of socio-economic position at the individual or aggregate level (e.g. Townsend index) would provide additional insight into embodiment processes.

The BHS was calculated based on 13 biomarkers covering five physiological systems. Although system-specific sub-scores and the BHS were normalised such that their value did not depend on the number of biomarkers they included, we cannot exclude the possibility that weaker associations involving systems with fewer biomarkers (e.g. kidney) may simply reflect a more imprecise description of the system. Sensitivity analyses using PCA scores as an unsupervised alternative to the BHS yielded similar conclusions, suggesting that the original construct of the score relying on biomarker dichotomisation did not affect the health-relevance of the score. However, effect-size estimates were smaller, which can be attributed to a scaling effect and to the proportion of variance explained by the first principal component, which ranged from 16 to 60% of the total variance of the biomarkers.

Throughout our analyses, the effect of the BHS was stronger than that of any of the system-specific sub-scores. This supports the use of a multisystem score such as the BHS that is able to capture complementary health-relevant information from each system. We included external cause mortality in our analyses as a negative control and found no association with the BHS and sub-scores. We cannot exclude that this lack of association may to some extent reflect the relatively lower statistical power (*N* = 315 deaths) for this outcome.

Overall, we found that increased values of the BHS were associated both with elevated mortality and CVD and cancer incidence. We show that this role is independent of (i) education as a marker of social determinants of health, and (ii) established (and potentially socially patterned) health risk factors.

In conclusion, we have shown that biological age, as measured by the BHS, is socially patterned and captures features of early life social embodiment. Independently of education, it complements health behaviours to better predict CVD incidence. Understanding the alternative mechanisms linking biological ageing markers, social determinants and adverse health outcomes, independently of behaviours, should be a research priority to identify novel targets for future policy interventions.

## Contributors

MC—H and BB, RV, and MKa contributed equally to the study. PE, MK-I and CD are joint last authors. MC—H, BB, RV, MKa, PE, MK-I and CD conceived the study and drafted the manuscript. MC—H, MKa, BB, VZ, JE, MW, and RC performed the statistical analyses. UK Biobank data were extracted, harmonised and analysed by IT, DP, DM, PV, SS, MKi provided insights into the study design, results interpretation and revised the manuscript. All authors revised the manuscript for important intellectual content and approved the submission of the manuscript. MC—H had full access to the data and takes responsibility for the integrity of the data and the accuracy of the data analysis and for the decision to submit for publication.

## Declaration of Competing Interest

Prof. Elliott is the director of the MRC Centre of Environment and Health (MR/L01341X/1 and MC/S019669/1) and has no conflict of interest to disclose. Prof Kivimäki reports grants from the Medical Research Council (MR/R024227/1), National Institute on ageing (NIA), US (R01AG056477), Academy of Finland (311,492) and Helsinki Institute of Life Science, outside the submitted work. All other authors do not have any interests to disclose.

## Appendix Supplementary materials

Image, application 1
