# Supplementary material for: Education, biological ageing, all-cause and cause-specific mortality and morbidity: UK biobank cohort study
Source: eClinicalMedicine. 2020 Nov 19;29-30:100658. doi: 10.1016/j.eclinm.2020.100658 (PMC7788440; doi:10.1016/j.eclinm.2020.100658)

Supplementary Information:

Education, Biological Ageing, and all cause, cause-specific mortality and Cancer, and Cardiovascular Disease incidence: UK Biobank Cohort Study

Prof Marc Chadeau-Hyam [a,b,†], PhD; Ms Barbara Bodinier, MSc [a,b,†]; Prof Roel Vermeulen, PhD [a,c, †]; Dr Maryam Karimi, PhD [a,b,†]; Dr Verena Zuber [a,b], PhD; Dr Raphaële Castagné, PhD [d]; Dr Joshua Elliott, MBBS [a,b]; Dr David Muller[a,b], PhD; Dr Dusan Petrovic [a,b], PhD; Mr Matthew D Whitaker [a,b]; Dr Silvia Stringhini, PhD [e,f]; Prof Ioanna Tzoulaki, PhD [a,b,g]; Prof Mika Kivimäki, FMedSci[h,i]; Prof Paolo Vineis, MD [a,b,j]; Prof Paul Elliott, MBBS [a,b,k,l,,‡]; Dr Michelle Kelly-Irving, PhD [c,‡] ; and Dr Cyrille Delpierre, PhD[c,‡]

[a] Department of Epidemiology and Biostatistics, School of Public Health, Imperial College London, London, UK
[b] MRC Centre for Environment and Health, Imperial College, London, UK
[c] Institute for Risk Assessment Sciences (IRAS), Utrecht University, Utrecht, The Netherlands
[d] LEASP, UMR 1027, Inserm-Université Toulouse III Paul Sabatier, Toulouse, France
[e] University Centre for General Medicine and Public Health (UNISANTE), Lausanne University, Lausanne, Switzerland
[f] Unit of Population Epidemiology, Department of Primary Care, Geneva University Hospitals, Geneva, Switzerland
[g] Department of Hygiene and Epidemiology, University of Ioannina Medical School, Ioannina, Greece
[h] Clinicum, Faculty of Medicine, University of Helsinki, Helsinki, Finland
[i] Department of Epidemiology and Public Health, University College London, London, UK
[j] Italian Institute for Genomic Medicine IIGM, Torino, Italy
[k] National Institute for Health Research, Biomedical Research Centre, Imperial College London, London, UK
[l] Health Data Research UK London at Imperial College London, London, London, UK
[†] Joint first authors
[‡] Joint last authors

Address for Correspondence:

Dr Marc Chadeau-Hyam
Department of Epidemiology and Biostatistics, School of Public Health,
St Mary's Hospital, Norfolk Place
London, W21PG, UK
E-mail: m.chadeau@imperial.ac.uk

Supplementary Methods:

1. Exploring the BHS and its gradients

We explore age and education-related gradients in the BHS and system-specific sub-scores in men and women separately. For each age group and gender, we calculated the BHS values in the low, intermediate, and high education groups, and tested for differences in these means using a Student's T-test. We also tested if these three values by age group and gender were supportive of a trend across education groups using a non-parametric Kruskal-Wallis rank test.

Using a linear model, we investigated if BHS differentials across SEP groups were driven by established health risk factors. For a given individual i, in a given age group a, a given gender g and a given SEP group, we investigated the contribution of potential confounders to social gradients in the BHS by using the following linear model, for each age group and gender separately (i.e. pooling data across SEP groups):

$$BHS_{i,a,g} = \alpha + \beta_{a,g}^{SEP_i} \times SEP_i + \beta_{a,g}^{FE} \times FE_i + \varepsilon_i, \text{ where}$$

FE_i combines the observed values of the set of potential confounders for individual i, and SEP_i is the SEP category individual i belongs to. The regression coefficients, $\beta_{a,g}^{Low}$, and $\beta_{a,g}^{Intermediate}$, are the estimates of the adjusted effect of SEP on the BHS for the low and intermediate SEP categories, respectively, and can be interpreted as the average difference in BHS in the low and intermediate SEP groups compared to what is observed in the high SEP group, independently of the adjustment variables.

We adopted a sequential adjustment approach first setting the unadjusted model as benchmark (Model A, not including any adjustment variable in FE), and sequentially adjusted the model for the following time-resolved covariates:

- Model B = Model A + Smoking, physical activity and alcohol consumption
- Model C = Model B + BMI
- Model D = Model C + Medicines and Treatments + Co-morbidities

2. Survival Analyses

The administrative censoring date was set to 1st of March 2016. The underlying causes of death were coded according to the International Classification of Diseases, 10th revision (ICD-10). Three broad categories of causes were considered: cardiovascular diseases (Supplementary Table 2), cancer (Supplementary Table 1) and external causes (ICD-10: V00-V99, W00-W99, X00-X99, Y00-Y99). Two incident health outcomes were considered: cardiovascular diseases and cancer diagnosis. These were obtained from hospitalisation registries, cancer registries, and for incident CVD, nurse administered questionnaires and were coded according to the International Classification of Diseases, 10th and 9th revisions (ICD-10 and ICD-9), non-cancer illness codes (Data-Coding 6 in UK Biobank), operation codes (Data-Coding 5) and the OPCS Classification of Interventions and Procedures (OPCS-4).

Cox proportional hazard models were used to estimate Hazard Ratios (HRs) and 95% Confidence Intervals (CIs) for the association between BHS and score-specific systems with (i) all-cause and cause-specific mortality and, (ii) cancer, and CVD incidence. Age was used as the time scale in the Cox models. The assessments were performed between 2006 and 2010 and hence, the Cox models accounted for left truncation induced by delayed entries. For cause-specific analyses, the models were fitted using a Cox model by censoring the participants who failed from competing causes.

Models were fitted in men and women and women separately. The first related education level (as predictor) and each of the health outcomes separately as a benchmark model. We then sequentially adjusted the model for the following variables:

- Behaviours (smoking, physical activity and alcohol consumption)
- BMI
- Numbers of co-morbidities and treatments
- BHS

Analyses were conducted to relate the BHS and each system specific sub-scores to health outcomes. The unadjusted model investigated associations between BHS/system-specific scores with all-cause and cause-specific mortality and cancer and CVD incident and the HRs were interpreted as the hazard corresponding to a 0.1 increase in BHS/scores. Models were

then sequentially adjusted for the following covariates introduced into the models as categorical variables:

- Education
- Behaviours (smoking, physical activity and alcohol consumption)
- BMI
- Numbers of comorbidities and treatments

Main analyses were conducted on the population excluding prevalent cases. Among the 474 clinical conditions informed within the UK Biobank dataset, we considered as comorbidities, any condition potentially affecting at least one of the five systems included in the BHS, and excluded the cancer (Supplementary Table 1) and CVD (Supplementary Table 2) outcomes of interest. Specifically, the comorbidities retained were:

1. Asthma (Non-cancer illness code, UK Biobank field 20002: 1111)
2. Arthritis (1311, 1464, 1538)
3. Emphysema (1113, 1472)
4. Hyperthyroidism (1225)
5. Hypothyroidism (1226)
6. Bronchitis (1412)
7. Liver condition (1604, 1136, 1158)
8. Diabetes (1220-1223, 1521)
9. Epilepsy (1264)
10. Depression (1286, 1291, 1531)
11. Kidney condition (1192, 1519, 1405)

We tested the validity of the proportional hazard assumption using Schoenfeld residuals.

3. Mendelian randomisation

To assess the possible causal nature of the observational association linking the BHS and the health outcomes, we adopted a one sample Mendelian randomisation approach. Specifically, our approach relied on a two-stage least squares model[1] and was conducted according to the following steps:

1. *Identification of the instrumental variables:*

   To identify relevant genetic instruments, we performed a genome-wide association study (GWAS) of the BHS adjusting for age, sex and the 10 first principal components capturing the UK Biobank population structure. This GWAS was done on the participants with full genotype data, who were healthy at recruitment (N=360,281). We adopted the same selection criteria as those defined in a recent study of the UK Biobank biomarkers data[2]. Genotype data included 672,345 SNPs of which 622,398 had a minor allele frequency greater than 0.01, and were included in the analysis. We selected genetic instruments for the BHS by using a series of univariate linear models regressing the genotype at each of these loci (as predictors) against the BHS (outcome). To select our genetic instruments of the BHS, we adopted a stringent pruning strategy which had recently been proposed to analyse UK Biobank biomarkers data[2], and relied on (i) a clumping step targeting SNPs within the genetic regions (10,000kB wide) of index SNPs (as defined by those with a p-value $<10^{-6}$), which were in high LD with the index SNPs ($r^2>0.1$), (ii) a thresholding step, selecting from the SNPs in the clumps those with p-values below the Bonferroni-corrected per-test significance level of 10^{-7}, and (iii) a tagging step removing correlated SNPs within those selected in (ii). When applied to the UK Biobank data, we identified 172 independent SNPs, which we use in the following as instrumental variables for the BHS. As sensitivity analyses, we performed the selection of instrumental variables using (i) the BOLT-LMM model as an alternative with relaxed normality assumptions, (ii) using a more stringent threshold of 10^{-8} on the p-values in the clumping step, and (iii) using a more stringent threshold for the pruning of SNPs in high LD ($r^2>0.01$).

2. *Estimate the instrumentally-explained BHS*

   Using a multivariable linear regression model setting the BHS as the outcome and the full set of 172 instrumental variables as predictors, we estimated, for each participant i, the part of the BHS explained by the full-set of instrumental variables ($\widehat{BHS}^i$) as:

$$\widehat{BHS}^i = \sum_{j=1}^{172} \beta_j \times g_j^i, \text{ where}$$

g_j^i, is the genotype of individual i for the genetic instrument j, and β_j, the effect size estimate for that instrument in the multivariable linear model.

3. *Estimating the 'causal effect' of the BHS*

   The second step of the two-stage least squares approach consists in regressing the instrumentally-explained exposure (here $\widehat{BHS}$) against the outcome of interest. To fit our survival analysis, and as recently proposed to analyse UK Biobank data[3], we used a Cox model to regress $\widehat{BHS}$ (as predictor) against each outcome (all-cause, cancer and CVD mortality, and cancer and CVD incidence) separately. The regression coefficient measuring the effect of the $\widehat{BHS}$ on survival can be interpreted as the 'causal effect', and its statistical significance is indicative of the causal nature of the link between the BHS and the outcome of interest. These analyses were conducted on the full population, and results are reported for a model corrected (i) for sex and the 10 first principal components capturing the UK Biobank population structure, and (ii) additionally for education.

4. *Estimating the 'causal effect' of the BHS conditionally on that of education.*

   To capture the potential mediating effect of education in the (possibly) causal link between the BHS and the outcomes of interest, we adopted a multivariable Mendelian randomisation (MVMR) approach[4]. Using the 172 instrumental variables from the BHS, we estimated the instrumentally-explained education level (as a continuous variable, i.e. assuming an additive effect of education), $\widehat{\text{Education}}$. Re-running the Cox model from step 3 including $\widehat{\text{Education}}$, we estimated the causal effect of the BHS, conditionally on that of the instrumentally-explained education level ($\widehat{\text{Education}}$). In that set-up, the attenuation of the causal effect of the BHS from the univariate model (step 3) to the MVRM model, would be indicative of a potential mediation effect of BHS on the outcome via education.

References

1. Burgess S, Small DS, Thompson SG. A review of instrumental variable estimators for Mendelian randomization. *Stat Methods Med Res* 2017; **26**(5): 2333-55.
2. Sinnott-Armstrong N, Tanigawa Y, Amar D, et al. Genetics of 38 blood and urine biomarkers in the UK Biobank. *bioRxiv* 2019: 660506.
3. Sun Y-Q, Burgess S, Staley JR, et al. Body mass index and all cause mortality in HUNT and UK Biobank studies: linear and non-linear mendelian randomisation analyses. *BMJ* 2019; **364**: l1042.
4. Sanderson E, Davey Smith G, Windmeijer F, Bowden J. An examination of multivariable Mendelian randomization in the single-sample and two-sample summary data settings. *Int J Epidemiol* 2018.

Supplementary Table 1: Definition of cancer cases in our study, for each disease ICD codes we report the number of deaths and incident cases during the follow-up.

	ICD-10	#Deaths	Incidence
Lip, oral cavity and pharynx	C00-C14	49	365
Digestive organs	C15-C26	1358	3,268
Respiratory and intrathoracic organs	C30-C39	795	1,149
Bone and articular cartilage	C40-C41	9	23
Skin	C43-C44	54	8,568
Mesothelial and soft tissue	C45-C49	186	321
Breast	C50	160	3,958
Female genital organs	C51-C58	223	1,082
Male genital organs	C60-C63	155	3,828
Urinary tract	C64-C68	211	1,087
Eye, brain and other parts of central nervous system	C69-C72	274	361
Thyroid and other endocrine glands	C73-C75	12	177
Malignant neoplasms of ill-defined, secondary and unspecified sites	C76-C80	211	551
Malignant neoplasms, stated or presumed to be primary, of lymphoid, haematopoietic and related tissue	C81-C96	203	1,484
Malignant neoplasms of independent (primary) multiple sites	C97		19
In situ neoplasms	D00-D09		2,348
Benign neoplasms	D10-D36	5	13,779
Neoplasms of uncertain or unknown behaviour	D37-D48	11	1,404
	**Total count:**	**3,915**	**43,772**

Supplementary Table 2: Definition of CVD cases in our study, for each disease ICD codes we report the number of deaths and incident cases during the follow-up.

			#Deaths	Incidence
**ICD-10**	Transient cerebral ischemic attacks and related syndromes	G45	1	899
	Ischemic heart diseases	I20-I25	731	8,824
	Cerebral infarction	I63	36	1,521
	Stroke	I64	72	228
	Cerebral atherosclerosis	I67.2		7
	Cerebrovascular disease	I67.9	6	28
**OPCS-4**	Heart	K		41
**Non-cancer illness code (UK Biobank field 20002)**	Angina	1074		14
	Heart attack/myocardial infarction	1075		3
	Transient ischaemic attack	1082		15
	Ischaemic stroke	1583		2
**Operation code (UK Biobank field 20004)**	Coronary angioplasty	1070		11
	Other arterial surgery/revascularisation procedures	1071		9
	Carotid artery surgery/endarterectomy	1105		2
	Carotid artery angioplasty +/- stent	1109		2
	Coronary angiogram	1514		47
		Total count:	846	11,653

Supplementary Table 3: UK Biobank study population distribution across age groups.

	< 50 years old	50-64 years old	> 64 years old	***Total***
Males	46,127	96,930	28,136	**171,193**
Females	51,726	113,793	30,036	**195,555**
Total	**97,853**	**210,723**	**58,172**	***366,748***

Supplementary Table 4: Number of deaths (A) and incident pathologies (B) recorded in UK Biobank up to March 1st, 2016 (in parenthesis are the corresponding numbers for the population selected in the present analysis). Results are presented in men and women separately and for all-cause and causx10-specific mortality (A) including cancer, cardiovascular diseases, and external causes. Numbers for incident pathologies are restricted to cancer and CVD (B)

A- Mortality

	All-cause	Cancer[1]	CVD[2]	External causes[3]	Other causes[4]
Males	8,735 (4,428)	4,316 (2,225)	1,547 (681)	328 (220)	2,544 (1,302)
Females	5,661 (2,716)	3,698 (1,689)	352 (165)	160 (95)	1,451 (767)
Total	***14,396 (7,144)***	***8,014 (3,914)***	***1,899 (846)***	***488 (315)***	***3,995 (2,069)***

[1] Cancer mortality events include all cancer sites as defined in Supplementary Table 1
[2] Cardiovascular mortality events include the most severe outcomes as defined in Supplementary Table 2
[3] External causes include accidents, suicide; ICD10: I00-I99 ICD10: V00-V99, W00-W99, X00-X99, Y00-Y99
[4] Oher causes include any cause not listed in 1-3.

B- Incident Pathologies

	Cancer[1]	CVD[2]
Males	26,123 (20,962)	10,114 (7,925)
Females	26,320 (22,810)	5,539 (3,728)
Total	***52,443 (43,772)***	***15,653 (11,653)***

[1] Cancer incident events include all cancer sites as defined in Supplementary Table 1
[2] Cardiovascular incident events include the outcomes defined in Supplementary Table 2

Supplementary Table 5. Mean and standard deviation of the Biological Health Score (BHS) calculated by age group. Results are presented for each gender and each group of the categorical covariates. Differences in BHS across covariate categories (i) within each age group and (ii) across all age groups (last column) were investigated using a Kruskal Wallis rank test and the corresponding p-values are reported. All tests yielded p-values $<10^{-16}$ and these are not presented in the table. Study population was restricted to healthy participants at enrolment.

	**Age group**			**Total**
	<50 (N=97,853) Mean (s.d)	50-64 (N=210,723) Mean (s.d)	>64 (N=58,172) Mean (s.d)	(n=366,748) Mean (s.d)
**Gender**				
Male	0.222 (0.165)	0.217 (0.157)	0.211 (0.150)	0.217 (0.158)
Female	0.239 (0.174)	0.231 (0.168)	0.226 (0.159)	0.232 (0.168)
**Education**				
Low	0.268 (0.177)	0.252 (0.169)	0.234 (0.159)	0.248 (0.167)
Intermediate	0.243 (0.173)	0.231 (0.165)	0.219 (0.154)	0.233 (0.166)
High	0.210 (0.163)	0.204 (0.156)	0.201 (0.149)	0.206 (0.157)
**Comorbidities**				
None	0.229 (0.170)	0.225 (0.163)	0.219 (0.155)	0.225 (0.164)
One or more	0.237 (0.170)	0.223 (0.162)	0.217 (0.153)	0.226 (0.163)
**Number of treatments**				
None	0.215 (0.163)	0.204 (0.156)	0.199 (0.150)	0.207 (0.158)
One	0.239 (0.170)	0.226 (0.162)	0.213 (0.153)	0.228 (0.163)
Two or more	0.272 (0.181)	0.255 (0.169)	0.235 (0.157)	0.253 (0.169)
**Smoking**				
Never	0.228 (0.169)	0.219 (0.163)	0.214 (0.154)	0.221 (0.163)
Yes	0.236 (0.171)	0.231 (0.164)	0.223 (0.156)	0.231 (0.164)
**Sports activity**				
At least one sport	0.219 (0.165)	0.214 (0.159)	0.210 (0.153)	0.215 (0.160)
None	0.257 (0.178)	0.243 (0.168)	0.231 (0.157)	0.244 (0.169)
**Alcohol consumption**				
Non-drinker	0.258 (0.177)	0.247 (0.167)	0.240 (0.161)	0.248 (0.168)
Social drinker	0.247 (0.174)	0.245 (0.170)	0.234 (0.157)	0.244 (0.169)
Moderate drinker	0.223 (0.167)	0.221 (0.162)	0.219 (0.155)	0.221 (0.163)
Daily drinker	0.222 (0.167)	0.214 (0.159)	0.208 (0.152)	0.215 (0.160)
**Body Mass index (Kg/m^2)**				
Below 25	0.169 (0.137)	0.163 (0.133)	0.169 (0.132)	0.165 (0.134)
25 and below 30	0.231 (0.160)	0.223 (0.155)	0.218 (0.151)	0.224 (0.156)
30 and below 40	0.336 (0.181)	0.310 (0.173)	0.286 (0.165)	0.312 (0.175)
above 40	0.429 (0.182)	0.367 (0.177)	0.341 (0.166)	0.383 (0.180)

Supplementary Table 6 Mean [2.5-97.5% confidence interval of the mean] of the system specific sub-scores in men (A) and women (B), and for each age and Education group separately. Differences in mean system-specific sub-scores were tested using a Student's T-test in each age group and gender separately, setting the mean score observed for the high education group in that age group and gender as a reference. We report the corresponding p-values.

A. Men

Age	< 50 years old			50-64 years old			> 64 years old		
Education	Low	Intermediate	High	Low	Intermediate	High	Low	Intermediate	High
Metabolic	0.27 [0.28-0.29]<br>1.62×10^{-50}	0.24 [0.24-0.25]<br>1.24×10^{-47}	0.20 [0.21-0.21]	0.25 [0.25-0.26]<br>9.24×10^{-64}	0.24 [0.24-0.24]<br>3.36×10^{-59}	0.21 [0.21-0.21]	0.24 [0.25-0.26]<br>6.95×10^{-27}	0.23 [0.23-0.24]<br>2.69×10^{-13}	0.20 [0.21-0.21]
Cardiovascular	0.27 [0.28-0.29]<br>6.80×10^{-21}	0.26 [0.26-0.27]<br>6.47×10^{-50}	0.21 [0.22-0.22]	0.26 [0.26-0.27]<br>5.82×10^{-57}	0.24 [0.25-0.25]<br>2.35×10^{-47}	0.21 [0.22-0.22]	0.24 [0.25-0.26]<br>1.22×10^{-06}	0.24 [0.25-0.25]<br>2.32×10^{-04}	0.22 [0.23-0.24]
Inflammatory	0.29 [0.30-0.31]<br>2.42×10^{-61}	0.23 [0.24-0.24]<br>6.21×10^{-50}	0.19 [0.19-0.20]	0.26 [0.27-0.28]<br>3.79×10^{-162}	0.22 [0.23-0.23]<br>7.46×10^{-77}	0.18 [0.19-0.19]	0.23 [0.24-0.25]<br>3.16×10^{-33}	0.20 [0.21-0.22]<br>5.82×10^{-11}	0.18 [0.18-0.19]
Liver	0.25 [0.26-0.27]<br>8.30×10^{-23}	0.23 [0.23-0.24]<br>5.50×10^{-34}	0.19 [0.20-0.20]	0.22 [0.23-0.24]<br>1.23×10^{-19}	0.22 [0.23-0.23]<br>7.48×10^{-31}	0.20 [0.20-0.21]	0.23 [0.24-0.25]<br>4.32×10^{-10}	0.22 [0.23-0.24]<br>1.79×10^{-04}	0.20 [0.21-0.22]
Kidney	0.13 [0.15-0.16]<br>3.06×10^{-06}	0.19 [0.20-0.21]<br>4.01×10^{-04}	0.18 [0.19-0.19]	0.17 [0.17-0.18]<br>8.65×10^{-01}	0.18 [0.18-0.19]<br>2.24×10^{-02}	0.17 [0.17-0.18]	0.13 [0.14-0.15]<br>3.02×10^{-01}	0.13 [0.14-0.15]<br>6.11×10^{-01}	0.13 [0.14-0.15]

B. Women

Metabolic	0.31 [0.32-0.33]<br>4.37×10^{-76}	0.24 [0.24-0.25]<br>1.51×10^{-69}	0.20 [0.20-0.20]	0.27 [0.27-0.28]<br>2.17×10^{-236}	0.23 [0.23-0.24]<br>2.38×10^{-99}	0.20 [0.20-0.20]	0.25 [0.25-0.26]<br>2.88×10^{-46}	0.22 [0.23-0.23]<br>7.85×10^{-14}	0.19 [0.20-0.20]
Cardiovascular	0.27 [0.29-0.30]<br>1.37×10^{-16}	0.26 [0.26-0.27]<br>1.09×10^{-41}	0.22 [0.22-0.23]	0.27 [0.27-0.28]<br>3.55×10^{-76}	0.25 [0.25-0.26]<br>1.39×10^{-50}	0.22 [0.22-0.23]	0.23 [0.24-0.25]<br>1.62×10^{-01}	0.23 [0.24-0.25]<br>1.00×10^{-01}	0.23 [0.24-0.24]
Inflammatory	0.29 [0.30-0.32]<br>1.83×10^{-48}	0.23 [0.23-0.24]<br>1.56×10^{-56}	0.18 [0.19-0.19]	0.26 [0.26-0.27]<br>2.30×10^{-194}	0.21 [0.21-0.22]<br>5.30×10^{-69}	0.18 [0.18-0.18]	0.21 [0.22-0.23]<br>4.65×10^{-18}	0.19 [0.20-0.20]<br>1.41×10^{-03}	0.17 [0.18-0.19]
Liver	0.30 [0.31-0.33]<br>2.69×10^{-13}	0.27 [0.28-0.28]<br>5.39×10^{-14}	0.25 [0.25-0.26]	0.26 [0.26-0.27]<br>3.15×10^{-48}	0.24 [0.25-0.25]<br>4.46×10^{-30}	0.22 [0.22-0.23]	0.24 [0.25-0.26]<br>3.17×10^{-09}	0.23 [0.23-0.24]<br>1.10×10^{-02}	0.22 [0.22-0.23]
Kidney	0.19 [0.21-0.23]<br>4.87×10^{-02}	0.24 [0.24-0.25]<br>1.97×10^{-03}	0.23 [0.23-0.24]	0.23 [0.24-0.25]<br>2.11x10-07	0.23 [0.24-0.24]<br>1.78×10^{-06}	0.22 [0.22-0.23]	0.22 [0.24-0.25]<br>6.00x10-07	0.21 [0.22-0.24]<br>1.13×10^{-03}	0.19 [0.20-0.21]

Supplementary Table 7: Mean [2.5-97.5% confidence interval of the mean] contribution of education to the BHS by age group in men (A) and women (B) in the three different age groups. Effects are calculated for the unadjusted model and for models sequentially adjusting for behaviours and lifestyle factors, BMI, and co-morbidities and medical treatments. Within each age group, the difference between the effect estimate in the low and intermediate education groups and that of the high education group was tested using a Student's T-test. Corresponding p-values are reported in italics.

A. Men

	Unadjusted model	+ Behaviors	+ BMI	+ Medical
	β (se)<br>*p-value*	β (se)<br>*p-value*	β (se)<br>*p-value*	β (se)<br>*p-value*
**<50 years old (reference high Education, N=18,669)**				
low (N=1,837)	0.25 [0.25-0.26]<br>$3.96\text{x}10^{-55}$	0.23 [0.24-0.24]<br>$3.69\text{x}10^{-42}$	0.15 [0.15-0.16]<br>$6.81\text{x}10^{-20}$	0.14 [0.15-0.16]<br>$5.29\text{x}10^{-18}$
Intermediate (N=19,997)	0.23 [0.23-0.24]<br>$5.94\text{x}10^{-105}$	0.22 [0.22-0.22]<br>$1.67\text{x}10^{-96}$	0.14 [0.14-0.14]<br>$1.91\text{x}10^{-32}$	0.13 [0.14-0.14]<br>$2.47\text{x}10^{-30}$
**50-64 years old (reference high Education, N=36,283)**				
low (N=8,341)	0.24 [0.24-0.24]<br>$1.32\text{x}10^{-140}$	0.22 [0.22-0.23]<br>$1.42\text{x}10^{-98}$	0.15 [0.16-0.16]<br>$3.88\text{x}10^{-39}$	0.15 [0.15-0.15]<br>$1.66\text{x}10^{-33}$
Intermediate (N=30,867)	0.22 [0.22-0.23]<br>$5.93\text{x}10^{-120}$	0.21 [0.21-0.21]<br>$5.15\text{x}10^{-92}$	0.15 [0.15-0.15]<br>$1.19\text{x}10^{-32}$	0.14 [0.14-0.15]<br>$1.91\text{x}10^{-30}$
**>64 years old (reference high Education, N=7,970)**				
low (N=4,037)	0.22 [0.23-0.23]<br>$3.82\text{x}10^{-39}$	0.21 [0.21-0.22]<br>$5.96\text{x}10^{-27}$	0.16 [0.16-0.17]<br>$5.96\text{x}10^{-11}$	0.15 [0.16-0.16]<br>$3.78\text{x}10^{-10}$
Intermediate (N=6,952)	0.21 [0.21-0.22]<br>$4.73\text{x}10^{-16}$	0.20 [0.20-0.20]<br>$4.03\text{x}10^{-11}$	0.15 [0.16-0.16]<br>$9.27\text{x}10^{-05}$	0.15 [0.15-0.16]<br>$1.94\text{x}10^{-04}$

B. Women

	Unadjusted model	+ Behaviors	+ BMI	+ Medical
	β (se)<br>*p-value*	β (se)<br>*p-value*	β (se)<br>*p-value*	β (se)<br>*p-value*
**>50 years old (reference high Education, N=21,577)**				
low (N=1,287)	0.28 [0.29-0.29]<br>$2.29\text{x}10^{-54}$	0.27 [0.28-0.29]<br>$2.86\text{x}10^{-33}$	0.20 [0.21-0.21]<br>$1.54\text{x}10^{-14}$	0.19 [0.20-0.20]<br>$1.22\text{x}10^{-12}$
Intermediate (N=23,117)	0.25 [0.25-0.25]<br>$7.80\text{x}10^{-91}$	0.25 [0.26-0.26]<br>$2.85\text{x}10^{-64}$	0.19 [0.19-0.19]<br>$7.07\text{x}10^{-23}$	0.18 [0.18-0.18]<br>$5.20\text{x}10^{-20}$
**50-64 years old (reference high Education, N=37,827)**				
low (N=10,842)	0.26 [0.26-0.27]<br>$2.55\text{x}10^{-278}$	0.26 [0.27-0.27]<br>$9.22\text{x}10^{-183}$	0.19 [0.19-0.20]<br>$8.40\text{x}10^{-93}$	0.18 [0.18-0.18]<br>$2.15\text{x}10^{-75}$
Intermediate (N=41,338)	0.23 [0.24-0.24]<br>$8.73\text{x}10^{-138}$	0.24 [0.24-0.25]<br>$3.04\text{x}10^{-94}$	0.17 [0.18-0.18]<br>$6.97\text{x}10^{-42}$	0.17 [0.17-0.17]<br>$7.85\text{x}10^{-35}$
**>64 years old (reference high Education, N=6,754)**				
low (N=4,967)	0.24 [0.24-0.25]<br>$5.58\text{x}10^{-39}$	0.24 [0.24-0.25]<br>$3.57\text{x}10^{-20}$	0.18 [0.19-0.19]<br>$1.68\text{x}10^{-09}$	0.17 [0.18-0.18]<br>$3.57\text{x}10^{-08}$
Intermediate (N=8,651)	0.22 [0.22-0.23]<br>$1.10\text{x}10^{-11}$	0.22 [0.23-0.23]<br>$1.14\text{x}10^{-06}$	0.17 [0.18-0.18]<br>$1.51\text{x}10^{-03}$	0.17 [0.17-0.17]<br>$4.12\text{x}10^{-03}$

Supplementary Table 8. Mean and [2.5-97.5% confidence interval] of the hazard ratio measuring the association between all-cause mortality (N=7,144 deaths, 4,428 men, 2,716 women) and the BHS in men (top) and women (bottom). HRs are expressed as a risk change for 0.1 increase in the score and corresponding p-values are reported for an unadjusted model and models sequentially adjusting for education, behaviours, BMI, and numbers of comorbidities and treatments.

A. Men

		**Unadjusted model**		**+ Education**		**+ Behaviours**		**+ BMI**		**+ Medical**	
		HR [95% CI]	p_{val}	HR [95% CI]	p_{val}	HR [95% CI]	p_{val}	HR [95% CI]	p_{val}	HR [95% CI]	p_{val}
**BHS**		1.14 [1.12-1.16]	7.63x10^{-44}	1.13 [1.11-1.15]	6.86x10^{-38}	1.11 [1.09-1.13]	4.01x10^{-28}	1.11 [1.09-1.13]	1.21x10^{-26}	1.10 [1.08-1.13]	1.73x10^{-24}
**Education**	Low			1.63 [1.50-1.76]	7.47x10^{-31}	1.46 [1.34-1.58]	1.87x10^{-18}	1.46 [1.35-1.59]	7.45x10^{-19}	1.44 [1.32-1.57]	2.06x10^{-17}
	Intermediate			1.23 [1.14-1.32]	1.69x10^{-08}	1.16 [1.08-1.24]	6.65x10^{-05}	1.16 [1.08-1.25]	3.91x10^{-05}	1.16 [1.08-1.25]	5.72x10^{-05}
**Smoking status**	Ever					1.60 [1.51-1.71]	2.28x10^{-48}	1.61 [1.51-1.71]	8.93x10^{-49}	1.57 [1.48-1.68]	1.62x10^{-44}
**Physical activity**	None					1.40 [1.32-1.49]	1.78x10^{-28}	1.39 [1.31-1.48]	5.41x10^{-27}	1.36 [1.28-1.44]	1.66x10^{-23}
**Alcohol**	Non drinker					1.37 [1.20-1.55]	2.30x10^{-06}	1.36 [1.19-1.54]	3.74x10^{-06}	1.32 [1.16-1.51]	2.08x10^{-05}
	Moderate					0.88 [0.80-0.97]	1.05x10^{-02}	0.89 [0.81-0.98]	2.00x10^{-02}	0.91 [0.82-1.00]	4.76x10^{-02}
	Daily drinker					0.91 [0.84-0.99]	3.77x10^{-02}	0.93 [0.85-1.01]	9.91x10^{-02}	0.95 [0.87-1.04]	2.42x10^{-01}
**BMI**	25 to 30							0.78 [0.72-0.84]	2.36x10^{-11}	0.76 [0.71-0.82]	5.49x10^{-13}
	30 to 40							0.88 [0.81-0.96]	3.75x10^{-03}	0.83 [0.76-0.91]	2.78x10^{-05}
	≥ 40							1.64 [1.34-1.99]	1.14x10^{-06}	1.46 [1.20-1.78]	1.91x10^{-04}
**Co-morbidities**	≥ 1									1.17 [1.08-1.28]	1.38x10^{-04}
**Treatments**	1									1.01 [0.92-1.11]	8.66x10^{-01}
	≥ 2									1.40 [1.31-1.50]	1.33x10^{-22}

B. Women

**BHS**		1.09 [1.07-1.12]	8.38x10^{-16}	1.09 [1.07-1.11]	7.58x10-15	1.08 [1.05-1.10]	6.49x10^{-11}	1.08 [1.05-1.10]	5.96x10^{-10}	1.07 [1.05-1.10]	4.70x10^{-09}
**Education**	Low			1.19 [1.07-1.33]	1.27x10^{-03}	1.05 [0.94-1.17]	3.91x10^{-01}	1.06 [0.95-1.18]	3.12x10^{-01}	1.04 [0.93-1.16]	4.64x10^{-01}
	Intermediate			0.99 [0.91-1.09]	8.84x10^{-01}	0.94 [0.85-1.03]	1.57x10^{-01}	0.94 [0.86-1.03]	1.91x10^{-01}	0.93 [0.85-1.02]	1.42x10^{-01}
**Smoking status**	Ever					1.65 [1.52-1.78]	2.36x10^{-37}	1.65 [1.52-1.78]	2.76x10^{-37}	1.63 [1.51-1.76]	1.42x10^{-35}
**Physical activity**	None					1.24 [1.15-1.34]	2.12x10^{-08}	1.24 [1.15-1.34]	3.73x10^{-08}	1.23 [1.14-1.32]	1.80x10^{-07}
**Alcohol**	Non drinker					1.27 [1.12-1.45]	1.60x10^{-04}	1.27 [1.12-1.44]	2.23x10^{-04}	1.25 [1.10-1.42]	5.01x10^{-04}
	Moderate					0.86 [0.77-0.95]	4.53x10^{-03}	0.87 [0.78-0.96]	7.27x10^{-03}	0.88 [0.79-0.97]	1.28x10^{-02}
	Daily drinker					0.78 [0.71-0.86]	7.35x10^{-07}	0.79 [0.72-0.87]	1.61x10^{-06}	0.80 [0.72-0.88]	5.58x10^{-06}
**BMI**	25 to 30							0.88 [0.80-0.96]	3.69x10^{-03}	0.87 [0.79-0.95]	1.72x10^{-03}
	30 to 40							0.92 [0.82-1.02]	1.10x10^{-01}	0.89 [0.80-0.99]	3.27x10^{-02}
	≥ 40							1.41 [1.13-1.74]	1.83x10^{-03}	1.33 [1.07-1.64]	1.03x10^{-02}
**Co-morbidities**	≥ 1									1.09 [0.99-1.20]	7.34x10^{-02}
**Treatments**	1									1.03 [0.92-1.16]	5.69x10^{-01}
	≥ 2									1.26 [1.15-1.37]	3.09x10^{-07}

Supplementary Table 9 Mean and [2.5-97.5% confidence interval] of the hazard ratio measuring the association between cancer mortality (N=3,914 deaths, 2,225 men, 1,689 women) and the BHS in men (top) and women (bottom). HRs are expressed as a risk change for 0.1 increase in the score and corresponding p-values are reported for an unadjusted model and models sequentially adjusting for education, behaviours, BMI, and numbers of comorbidities and treatments.

A. Men

		**Unadjusted model**		**+ Education**		**+ Behaviours**		**+ BMI**		**+ Medical**	
		HR [95% CI]	p_{val}	HR [95% CI]	p_{val}	HR [95% CI]	p_{val}	HR [95% CI]	p_{val}	HR [95% CI]	p_{val}
**BHS**		1.11 [1.09-1.14]	1.00x10^{-16}	1.11 [1.08-1.13]	1.71x10^{-14}	1.09 [1.06-1.12]	6.58x10^{-11}	1.09 [1.06-1.12]	4.98x10^{-10}	1.09 [1.06-1.12]	7.89x10^{-10}
**Education**	Low			1.55 [1.38-1.74]	9.40x10^{-14}	1.40 [1.25-1.58]	1.58x10^{-08}	1.40 [1.25-1.58]	1.61x10^{-08}	1.40 [1.24-1.57]	2.32x10^{-08}
	Intermediate			1.18 [1.07-1.31]	1.19x10^{-03}	1.11 [1.01-1.23]	3.71x10^{-02}	1.12 [1.01-1.24]	3.51x10^{-02}	1.12 [1.01-1.23]	3.63x10^{-02}
**Smoking status**	Ever					1.72 [1.58-1.89]	5.06x10^{-32}	1.73 [1.58-1.89]	5.65x10^{-32}	1.72 [1.57-1.88]	2.91x10^{-31}
**Physical activity**	None					1.23 [1.13-1.34]	2.12x10^{-06}	1.22 [1.12-1.33]	3.35x10^{-06}	1.22 [1.12-1.33]	6.40x10^{-06}
**Alcohol**	Non drinker					1.28 [1.06-1.56]	1.19x10^{-02}	1.28 [1.06-1.56]	1.23x10^{-02}	1.27 [1.05-1.55]	1.48x10^{-02}
	Moderate					0.97 [0.84-1.11]	6.29x10^{-01}	0.97 [0.84-1.12]	6.70x10^{-01}	0.97 [0.85-1.12]	7.17x10^{-01}
	Daily drinker					0.99 [0.88-1.12]	9.16x10^{-01}	1.00 [0.88-1.13]	9.89x10^{-01}	1.01 [0.89-1.14]	9.20x10^{-01}
**BMI**	25 to 30							0.87 [0.78-0.97]	9.70x10^{-03}	0.87 [0.78-0.96]	7.80x10^{-03}
	30 to 40							0.98 [0.87-1.11]	7.54x10^{-01}	0.97 [0.86-1.10]	6.21x10^{-01}
	≥ 40							1.03 [0.72-1.49]	8.63x10^{-01}	1.01 [0.70-1.46]	9.53x10^{-01}
**Co-morbidities**	≥ 1									1.09 [0.97-1.23]	1.61x10^{-01}
**Treatments**	1									1.05 [0.93-1.19]	4.19x10^{-01}
	≥ 2									1.08 [0.98-1.19]	1.26x10^{-01}

B. Women

		**Unadjusted model**		**+ Education**		**+ Behaviours**		**+ BMI**		**+ Medical**	
**BHS**		1.07 [1.04-1.10]	8.54x10^{-06}	1.07 [1.04-1.10]	8.90x10^{-06}	1.06 [1.03-1.09]	1.17x10^{-04}	1.06 [1.03-1.09]	2.30x10^{-04}	1.06 [1.03-1.09]	2.22x10^{-04}
**Education**	Intermediate			1.02 [0.89-1.17]	7.89x10^{-01}	0.93 [0.81-1.07]	2.87x10^{-01}	0.93 [0.81-1.07]	3.06x10^{-01}	0.93 [0.81-1.07]	3.07x10^{-01}
	High			0.93 [0.83-1.04]	1.92x10^{-01}	0.89 [0.79-0.99]	4.06x10^{-02}	0.89 [0.79-1.00]	4.40x10^{-02}	0.89 [0.80-1.00]	4.50x10^{-02}
**Smoking status**	Ever					1.68 [1.52-1.85]	1.47x10^{-25}	1.68 [1.52-1.85]	1.47x10^{-25}	1.67 [1.52-1.84]	3.07x10^{-25}
**Physical activity**	None					1.08 [0.98-1.19]	1.07x10^{-01}	1.08 [0.98-1.19]	1.08x10^{-01}	1.08 [0.98-1.19]	1.12x10^{-01}
**Alcohol**	Non drinker					1.16 [0.98-1.38]	8.12x10^{-02}	1.16 [0.98-1.37]	8.40x10^{-02}	1.16 [0.98-1.37]	8.79x10^{-02}
	Moderate					0.90 [0.79-1.02]	1.08x10^{-01}	0.90 [0.79-1.03]	1.13x10^{-01}	0.90 [0.79-1.03]	1.19x10^{-01}
	Daily drinker					0.84 [0.75-0.95]	5.47x10^{-03}	0.84 [0.74-0.95]	5.89x10^{-03}	0.84 [0.75-0.95]	6.80x10^{-03}
**BMI**	25 to 30							0.95 [0.85-1.07]	4.13x10^{-01}	0.95 [0.85-1.07]	4.08x10^{-01}
	30 to 40							0.98 [0.85-1.12]	7.73x10^{-01}	0.98 [0.85-1.12]	7.68x10^{-01}
	≥ 40							1.04 [0.75-1.44]	8.16x10^{-01}	1.04 [0.75-1.44]	8.10x10^{-01}
**Co-morbidities**	≥ 1									1.10 [0.97-1.24]	1.37x10^{-01}
**Treatments**	1									1.01 [0.88-1.16]	8.90x10^{-01}
	≥ 2									1.00 [0.90-1.12]	9.91x10^{-01}

Supplementary Table 10. Mean and [2.5-97.5% confidence interval] of the hazard ratio measuring the association between cardiovascular mortality (N=846 deaths, 681 men, 165 women) and the BHS in men (top) and women (bottom). HRs are expressed as a risk change for 0.1 increase in the score and corresponding p-values are reported for an unadjusted model and models sequentially adjusting for education, behaviours, BMI, and numbers of comorbidities and treatments.

A. Men

		**Unadjusted model**		**+ Education**		**+ Behaviours**		**+ BMI**		**+ Medical**	
		HR [95% CI]	p_{val}	HR [95% CI]	p_{val}	HR [95% CI]	p_{val}	HR [95% CI]	p_{val}	HR [95% CI]	p_{val}
**BHS**		1.25 [1.20-1.31]	2.70x10^{-24}	1.25 [1.19-1.30]	6.64x10^{-23}	1.23 [1.18-1.29]	4.06x10^{-20}	1.23 [1.17-1.28]	1.07x10-17	1.22 [1.16-1.28]	8.80x10-
**Education**	Low			1.39 [1.12-1.72]	2.51x10^{-03}	1.23 [0.99-1.53]	6.08x10^{-02}	1.23 [0.99-1.53]	6.63x10^{-02}	1.20 [0.97-1.49]	9.94x10^{-02}
	Intermediate			1.18 [0.99-1.41]	6.56x10^{-02}	1.11 [0.93-1.33]	2.56x10^{-01}	1.11 [0.92-1.33]	2.68x10^{-01}	1.10 [0.92-1.32]	2.83x10^{-01}
**Smoking status**	Ever					1.51 [1.29-1.78]	3.66x10^{-07}	1.51 [1.29-1.77]	4.46x10^{-07}	1.47 [1.25-1.73]	2.39x10^{-06}
**Physical activity**	None					1.33 [1.14-1.55]	2.26x10^{-04}	1.32 [1.13-1.54]	3.53x10^{-04}	1.28 [1.10-1.50]	1.48x10^{-03}
**Alcohol**	Non drinker					1.20 [0.85-1.68]	2.96x10^{-01}	1.20 [0.85-1.68]	2.98x10^{-01}	1.16 [0.82-1.63]	3.96x10^{-01}
	Moderate					0.96 [0.76-1.22]	7.61x10^{-01}	0.97 [0.76-1.23]	7.95x10^{-01}	0.99 [0.78-1.26]	9.38x10^{-01}
	Daily drinker					0.84 [0.67-1.04]	1.07x10^{-01}	0.85 [0.68-1.05]	1.34x10^{-01}	0.87 [0.70-1.08]	2.13x10^{-01}
**BMI**	25 to 30							0.94 [0.77-1.14]	5.20x10^{-01}	0.91 [0.75-1.11]	3.75x10^{-01}
	30 to 40							1.01 [0.81-1.27]	9.17x10^{-01}	0.94 [0.75-1.18]	5.94x10^{-01}
	≥ 40							1.48 [0.88-2.51]	1.41x10^{-01}	1.28 [0.76-2.18]	3.56x10^{-01}
**Co-morbidities**	≥ 1									1.19 [0.97-1.47]	9.90x10^{-02}
**Treatments**	1									0.90 [0.70-1.16]	4.33x10^{-01}
	≥ 2									1.53 [1.29-1.82]	8.80x10^{-07}

B. Women

**BHS**		1.21 [1.11-1.31]	1.21x10^{-05}	1.19 [1.10-1.30]	4.72x10^{-05}	1.17 [1.07-1.27]	3.32x10^{-04}	1.17 [1.07-1.28]	7.00x10^{-04}	1.16 [1.06-1.27]	1.53x10^{-03}
**Education**	Low			2.09 [1.34-3.27]	1.25x10^{-03}	1.74 [1.10-2.74]	1.72x10^{-02}	1.76 [1.12-2.79]	1.48x10^{-02}	1.70 [1.07-2.68]	2.35x10^{-02}
	Intermediate			1.30 [0.86-1.98]	2.17x10^{-01}	1.19 [0.78-1.82]	4.07x10^{-01}	1.20 [0.79-1.83]	3.94x10^{-01}	1.18 [0.77-1.79]	4.52x10^{-01}
**Smoking status**	Ever					1.69 [1.24-2.31]	9.07x10^{-04}	1.69 [1.24-2.30]	9.40x10^{-04}	1.66 [1.22-2.26]	1.43x10^{-03}
**Physical activity**	None					1.29 [0.95-1.76]	1.01x10^{-01}	1.28 [0.94-1.75]	1.16x10^{-01}	1.25 [0.92-1.71]	1.54x10^{-01}
**Alcohol**	Non drinker					1.30 [0.82-2.07]	2.67x10^{-01}	1.28 [0.81-2.04]	2.95x10^{-01}	1.25 [0.79-1.99]	3.44x10^{-01}
	Moderate					0.76 [0.50-1.15]	2.01x10^{-01}	0.77 [0.51-1.17]	2.26x10^{-01}	0.79 [0.52-1.19]	2.57x10^{-01}
	Daily drinker					0.61 [0.41-0.91]	1.62x10^{-02}	0.62 [0.41-0.93]	2.00x10^{-02}	0.63 [0.42-0.94]	2.52x10^{-02}
**BMI**	25 to 30							0.89 [0.62-1.29]	5.55x10^{-01}	0.87 [0.60-1.26]	4.59x10^{-01}
	30 to 40							0.77 [0.49-1.20]	2.49x10^{-01}	0.71 [0.45-1.12]	1.37x10^{-01}
	≥ 40							2.12 [1.07-4.22]	3.22x10^{-02}	1.83 [0.91-3.65]	8.84x10^{-02}
**Co-morbidities**	≥ 1									0.82 [0.53-1.25]	3.55x10^{-01}
**Treatments**	1									1.24 [0.74-2.07]	4.12x10^{-01}
	≥ 2									1.84 [1.26-2.68]	1.66x10^{-03}

Supplementary Table 11. Mean and [2.5-97.5% confidence interval] of the hazard ratio measuring the association between external-cause mortality (N=315 deaths, 220 men, 95 women) and the BHS in men (top) and women (bottom). HRs are expressed as a risk change for 0.1 increase in the score and corresponding p-values are reported for an unadjusted model and models sequentially adjusting for education, behaviours, BMI, and numbers of comorbidities and treatments.

A. Men

		Unadjusted model		+ Education		+ Behaviours		+ BMI		+ Medical	
		HR [95% CI]	p_{val}	HR [95% CI]	p_{val}	HR [95% CI]	p_{val}	HR [95% CI]	p_{val}	HR [95% CI]	p_{val}
**BHS**		0.99 [0.91-1.08]	8.49×10^{-01}	0.98 [0.90-1.06]	6.00×10^{-01}	0.97 [0.89-1.06]	4.81×10^{-01}	1.00 [0.92-1.10]	9.65×10^{-01}	1.00 [0.91-1.09]	9.22×10^{-01}
**Education**	Low			2.13 [1.44-3.15]	1.64×10^{-04}	2.05 [1.37-3.05]	4.51×10^{-04}	2.13 [1.42-3.18]	2.25×10^{-04}	2.09 [1.40-3.13]	3.18×10^{-04}
	Intermediate			1.46 [1.06-2.00]	2.00×10^{-02}	1.44 [1.05-1.99]	2.48×10^{-02}	1.49 [1.08-2.05]	1.48×10^{-02}	1.50 [1.09-2.07]	1.35×10^{-02}
**Smoking status**	Ever					1.25 [0.95-1.64]	1.11×10^{-01}	1.26 [0.96-1.66]	9.30×10^{-02}	1.22 [0.93-1.61]	1.47×10^{-01}
**Physical activity**	None					1.19 [0.90-1.57]	2.17×10^{-01}	1.21 [0.91-1.59]	1.87×10^{-01}	1.16 [0.88-1.53]	2.94×10^{-01}
**Alcohol**	Non drinker					2.43 [1.34-4.44]	3.64×10^{-03}	2.40 [1.32-4.37]	4.26×10^{-03}	2.28 [1.25-4.15]	7.18×10^{-03}
	Moderate					1.54 [0.95-2.50]	7.81×10^{-02}	1.56 [0.96-2.52]	7.25×10^{-02}	1.60 [0.99-2.60]	5.49×10^{-02}
	Daily drinker					1.58 [1.01-2.48]	4.70×10^{-02}	1.57 [1.00-2.47]	4.98×10^{-02}	1.64 [1.04-2.58]	3.18×10^{-02}
**BMI**	25 to 30							0.64 [0.47-0.88]	5.09×10^{-03}	0.64 [0.47-0.87]	4.13×10^{-03}
	30 to 40							0.64 [0.43-0.94]	2.34×10^{-02}	0.61 [0.41-0.90]	1.25×10^{-02}
	≥ 40							0.51 [0.12-2.12]	3.56×10^{-01}	0.46 [0.11-1.89]	2.79×10^{-01}
**Co-morbidities**	≥ 1									1.82 [1.32-2.50]	2.52×10^{-04}
**Treatments**	1									0.75 [0.47-1.18]	2.11×10^{-01}
	≥ 2									1.51 [1.12-2.05]	7.09×10^{-03}

B. Women

		Unadjusted model		+ Education		+ Behaviours		+ BMI		+ Medical	
**BHS**		0.94 [0.83-1.07]	3.51×10^{-01}	0.94 [0.83-1.06]	3.21×10^{-01}	0.93 [0.82-1.06]	2.80×10^{-01}	0.96 [0.84-1.10]	5.33×10^{-01}	0.96 [0.84-1.10]	5.31×10^{-01}
**Education**	Low			1.19 [0.61-2.32]	6.00×10^{-01}	1.07 [0.55-2.11]	8.34×10^{-01}	1.10 [0.56-2.17]	7.76×10^{-01}	1.11 [0.56-2.18]	7.72×10^{-01}
	Intermediate			1.19 [0.75-1.88]	4.53×10^{-01}	1.14 [0.72-1.81]	5.76×10^{-01}	1.16 [0.73-1.84]	5.33×10^{-01}	1.16 [0.73-1.84]	5.21×10^{-01}
**Smoking status**	Ever					1.60 [1.06-2.40]	2.45×10^{-02}	1.61 [1.07-2.42]	2.24×10^{-02}	1.59 [1.06-2.39]	2.66×10^{-02}
**Physical activity**	None					1.46 [0.97-2.19]	7.01×10^{-02}	1.49 [0.99-2.23]	5.75×10^{-02}	1.48 [0.98-2.22]	6.24×10^{-02}
**Alcohol**	Non drinker					1.77 [0.82-3.82]	1.44×10^{-01}	1.76 [0.82-3.79]	1.50×10^{-01}	1.72 [0.80-3.72]	1.64×10^{-01}
	Moderate					1.65 [0.93-2.93]	8.93×10^{-02}	1.62 [0.91-2.89]	9.98×10^{-02}	1.64 [0.92-2.93]	9.15×10^{-02}
	Daily drinker					1.29 [0.73-2.27]	3.79×10^{-01}	1.25 [0.71-2.20]	4.48×10^{-01}	1.26 [0.72-2.23]	4.20×10^{-01}
**BMI**	25 to 30							0.82 [0.52-1.30]	3.91×10^{-01}	0.81 [0.51-1.29]	3.80×10^{-01}
	30 to 40							0.69 [0.37-1.29]	2.48×10^{-01}	0.68 [0.36-1.28]	2.31×10^{-01}
	≥ 40							0.83 [0.19-3.57]	8.05×10^{-01}	0.80 [0.19-3.46]	7.67×10^{-01}
**Co-morbidities**	≥ 1									1.46 [0.90-2.36]	1.22×10^{-01}
**Treatments**	1									0.51 [0.25-1.05]	6.70×10^{-02}
	≥ 2									1.04 [0.67-1.64]	8.50×10^{-01}

Supplementary Table 12 Mean and [2.5-97.5% confidence interval] of the hazard ratio measuring the association between cancer incidence (N=43,772 cases, 20,962 men, 22,810 women) and the BHS in men (top) and women (bottom). HRs are expressed as a risk change for 0.1 increase in the score and corresponding p-values are reported for an unadjusted model and models sequentially adjusting for education, behaviours, BMI, and numbers of comorbidities and treatments.

A. Men

		**Unadjusted model**		**+ Education**		**+ Behaviours**		**+ BMI**		**+ Medical**	
		HR [95% CI]	p_{val}	HR [95% CI]	p_{val}	HR [95% CI]	p_{val}	HR [95% CI]	p_{val}	HR [95% CI]	p_{val}
**BHS**		1.02 [1.01-1.03]	1.01×10^{-04}	1.01 [1.01-1.02]	9.01×10^{-04}	1.01 [1.00-1.02]	4.47×10^{-03}	1.01 [1.00-1.02]	2.02×10^{-01}	1.00 [0.99-1.01]	4.22×10^{-01}
**Education**	Low			1.14 [1.10-1.19]	1.49×10^{-11}	1.13 [1.09-1.18]	2.42×10^{-09}	1.12 [1.08-1.17]	2.95×10^{-08}	1.11 [1.07-1.16]	1.90×10^{-07}
	Intermediate			1.05 [1.02-1.09]	8.06×10^{-04}	1.04 [1.01-1.08]	6.57×10^{-03}	1.04 [1.01-1.07]	1.84×10^{-02}	1.04 [1.00-1.07]	2.94×10^{-02}
**Smoking status**	Ever					1.12 [1.09-1.15]	2.56×10^{-15}	1.11 [1.08-1.15]	3.15×10^{-14}	1.11 [1.08-1.14]	1.22×10^{-12}
**Physical activity**	None					1.00 [0.98-1.03]	7.72×10^{-01}	1.00 [0.97-1.03]	9.60×10^{-01}	0.99 [0.96-1.02]	5.76×10^{-01}
**Alcohol**	Non drinker					0.95 [0.89-1.02]	1.75×10^{-01}	0.95 [0.89-1.02]	1.97×10^{-01}	0.95 [0.88-1.02]	1.35×10^{-01}
	Moderate					1.01 [0.97-1.06]	6.13×10^{-01}	1.01 [0.97-1.06]	6.06×10^{-01}	1.02 [0.97-1.06]	4.69×10^{-01}
	Daily drinker					1.02 [0.98-1.06]	3.53×10^{-01}	1.02 [0.98-1.07]	2.61×10^{-01}	1.03 [0.99-1.07]	1.78×10^{-01}
**BMI**	25 to 30							1.04 [1.01-1.08]	2.12×10^{-02}	1.03 [1.00-1.07]	8.38×10^{-02}
	30 to 40							1.10 [1.06-1.15]	2.84×10^{-06}	1.08 [1.03-1.12]	5.88×10^{-04}
	≥ 40							1.16 [1.02-1.32]	2.06×10^{-02}	1.11 [0.97-1.26]	1.21×10^{-01}
**Co-morbidities**	≥ 1									0.99 [0.95-1.03]	5.38×10^{-01}
**Treatments**	1									1.09 [1.05-1.13]	2.45×10^{-05}
	≥ 2									1.16 [1.12-1.19]	1.73×10^{-19}

B. Women

**BHS**		1.02 [1.01-1.03]	1.07×10^{-05}	1.02 [1.01-1.03]	1.30×10^{-05}	1.02 [1.01-1.02]	6.45×10^{-05}	1.01 [1.00-1.02]	9.03×10^{-02}	1.01 [1.00-1.01]	2.01×10^{-01}
**Education**	Low			1.01 [0.97-1.05]	5.48×10^{-01}	0.99 [0.95-1.03]	7.37×10^{-01}	0.99 [0.95-1.03]	5.20×10^{-01}	0.98 [0.94-1.02]	3.34×10^{-01}
	Intermediate			0.99 [0.97-1.02]	6.80×10^{-01}	0.98 [0.96-1.01]	3.08×10^{-01}	0.98 [0.95-1.01]	1.97×10^{-01}	0.98 [0.95-1.01]	1.31×10^{-01}
**Smoking status**	Ever					1.11 [1.09-1.14]	1.46×10^{-15}	1.11 [1.08-1.14]	7.92×10^{-15}	1.11 [1.08-1.14]	3.47×10^{-14}
**Physical activity**	None					1.05 [1.02-1.08]	5.82×10^{-04}	1.04 [1.01-1.07]	4.26×10^{-03}	1.04 [1.01-1.06]	9.04×10^{-03}
**Alcohol**	Non drinker					0.99 [0.94-1.04]	6.20×10^{-01}	0.99 [0.94-1.04]	6.48×10^{-01}	0.98 [0.94-1.04]	5.55×10^{-01}
	Moderate					1.01 [0.98-1.05]	5.31×10^{-01}	1.02 [0.98-1.06]	2.96×10^{-01}	1.02 [0.99-1.06]	2.29×10^{-01}
	Daily drinker					0.98 [0.95-1.02]	2.88×10^{-01}	1.00 [0.96-1.03]	8.00×10^{-01}	1.00 [0.97-1.03]	9.57×10^{-01}
**BMI**	25 to 30							1.04 [1.01-1.07]	1.12×10^{-02}	1.04 [1.01-1.07]	2.16×10^{-02}
	30 to 40							1.09 [1.05-1.14]	4.05×10^{-06}	1.08 [1.04-1.12]	6.02×10^{-05}
	≥ 40							1.27 [1.16-1.38]	8.06×10^{-08}	1.24 [1.14-1.35]	1.38×10^{-06}
**Co-morbidities**	≥ 1									0.98 [0.94-1.01]	1.78×10^{-01}
**Treatments**	1									1.04 [1.01-1.08]	2.43×10^{-02}
	≥ 2									1.10 [1.06-1.13]	1.57×10^{-09}

Supplementary Table 13 Mean and [2.5-97.5% confidence interval] of the hazard ratio measuring the association between cardiovascular disease incidence (N=11,653 cases, 7,925 men, 3,728 women) and the BHS in men (top) and women (bottom). HRs are expressed as a risk change for 0.1 increase in the score and corresponding p-values are reported for an unadjusted model and models sequentially adjusting for education, behaviours, BMI, and numbers of comorbidities and treatments.

A. Men

		**Unadjusted model**		**+ Education**		**+ Behaviours**		**+ BMI**		**+ Medical**	
		HR [95% CI]	p_{val}	HR [95% CI]	p_{val}	HR [95% CI]	p_{val}	HR [95% CI]	p_{val}	HR [95% CI]	p_{val}
**BHS**		1.15 [1.13-1.16]	1.28×10^{-93}	1.14 [1.13-1.16]	2.63×10^{-84}	1.13 [1.12-1.15]	8.71×10^{-76}	1.11 [1.10-1.13]	3.87×10^{-50}	1.11 [1.09-1.12]	2.69×10^{-46}
**Education**	Low			1.39 [1.30-1.48]	6.59×10^{-24}	1.29 [1.20-1.37]	3.71×10^{-14}	1.26 [1.18-1.34]	7.86×10^{-12}	1.24 [1.16-1.32]	1.47×10^{-10}
	Intermediate			1.24 [1.17-1.30]	8.76×10^{-16}	1.19 [1.13-1.26]	3.40×10^{-11}	1.17 [1.11-1.24]	2.06×10^{-09}	1.17 [1.11-1.23]	7.48×10^{-09}
**Smoking status**	Ever					1.20 [1.15-1.26]	5.60×10^{-15}	1.18 [1.13-1.24]	3.63×10^{-13}	1.17 [1.12-1.22]	2.47×10^{-11}
**Physical activity**	None					1.12 [1.07-1.17]	1.01×10^{-06}	1.11 [1.06-1.16]	1.41×10^{-05}	1.09 [1.04-1.14]	2.26×10^{-04}
**Alcohol**	Non drinker					1.14 [1.03-1.26]	9.90×10^{-03}	1.15 [1.04-1.27]	6.05×10^{-03}	1.13 [1.03-1.25]	1.37×10^{-02}
	Moderate					0.91 [0.85-0.97]	5.06×10^{-03}	0.90 [0.84-0.97]	4.30×10^{-03}	0.91 [0.85-0.98]	9.98×10^{-03}
	Daily drinker					0.79 [0.74-0.84]	8.27×10^{-14}	0.80 [0.75-0.85]	7.21×10^{-13}	0.80 [0.75-0.85]	6.06×10^{-12}
**BMI**	25 to 30							1.18 [1.11-1.25]	7.27×10^{-08}	1.15 [1.09-1.22]	2.38×10^{-06}
	30 to 40							1.36 [1.27-1.46]	4.90×10^{-19}	1.29 [1.21-1.39]	1.36×10^{-13}
	≥ 40							1.31 [1.09-1.59]	4.53×10^{-03}	1.19 [0.99-1.44]	6.61×10^{-02}
**Co-morbidities**	≥ 1									0.96 [0.89-1.02]	1.81×10^{-01}
**Treatments**	1									1.19 [1.12-1.27]	1.34×10^{-07}
	≥ 2									1.33 [1.27-1.40]	1.89×10^{-28}

B. Women

		**Unadjusted model**		**+ Education**		**+ Behaviours**		**+ BMI**		**+ Medical**	
**BHS**		1.17 [1.15-1.19]	6.84×10^{-65}	1.16 [1.14-1.18]	1.27×10^{-59}	1.15 [1.13-1.17]	4.37×10^{-50}	1.12 [1.10-1.15]	4.71×10^{-32}	1.11 [1.09-1.14]	2.17×10^{-27}
**Education**	Low			1.40 [1.27-1.54]	4.02×10^{-12}	1.25 [1.13-1.38]	6.87×10^{-06}	1.23 [1.11-1.35]	4.13×10^{-05}	1.18 [1.07-1.30]	7.51×10^{-04}
	Intermediate			1.24 [1.14-1.34]	1.64×10^{-07}	1.17 [1.08-1.27]	9.69×10^{-05}	1.16 [1.07-1.26]	3.23×10^{-04}	1.14 [1.05-1.23]	1.71×10^{-03}
**Smoking status**	Ever					1.31 [1.22-1.39]	1.18×10^{-15}	1.30 [1.21-1.38]	8.04×10^{-15}	1.27 [1.19-1.36]	5.80×10^{-13}
**Physical activity**	None					1.12 [1.05-1.20]	4.91×10^{-04}	1.10 [1.03-1.18]	3.33×10^{-03}	1.08 [1.01-1.15]	2.08×10^{-02}
**Alcohol**	Non drinker					1.18 [1.06-1.31]	2.29×10^{-03}	1.19 [1.07-1.32]	1.65×10^{-03}	1.16 [1.04-1.29]	6.51×10^{-03}
	Moderate					0.87 [0.79-0.94]	1.11×10^{-03}	0.88 [0.81-0.96]	3.68×10^{-03}	0.90 [0.82-0.98]	1.27×10^{-02}
	Daily drinker					0.71 [0.65-0.77]	2.22×10^{-16}	0.73 [0.67-0.79]	1.60×10^{-13}	0.75 [0.69-0.81]	5.63×10^{-12}
**BMI**	25 to 30							1.20 [1.11-1.30]	9.72×10^{-06}	1.17 [1.08-1.26]	1.34×10^{-04}
	30 to 40							1.31 [1.20-1.44]	7.44×10^{-09}	1.23 [1.12-1.35]	1.33×10^{-05}
	≥ 40							1.45 [1.20-1.76]	1.53×10^{-04}	1.28 [1.05-1.55]	1.27×10^{-02}
**Co-morbidities**	≥ 1									0.96 [0.88-1.05]	3.79×10^{-01}
**Treatments**	1									1.29 [1.17-1.43]	6.73×10^{-07}
	≥ 2									1.69 [1.57-1.83]	3.13×10^{-41}

Supplementary Table 14 Summary statistics of the genome-wide association study of the BHS adjusted for age, sex and the first 10 principal components. The effect size, standard error and p-value are reported for the 172 pruned variants used as genetic instruments of the BHS.

Chromosome	Position	ID	Effect allele	β	se	p-value
1	16505908	rs4661718	C	-3.67e-03	3.98e-04	2.82e-20
1	16742820	rs11260752	C	2.19e-03	3.94e-04	2.58e-08
1	54222571	rs1183394	A	-2.37e-03	6.01e-04	7.85e-05
1	63025942	rs2131925	G	-3.02e-03	4.04e-04	7.49e-14
1	66102257	rs1805096	A	-2.44e-03	3.99e-04	9.01e-10
1	91533297	rs165316	G	-3.49e-03	4.87e-04	7.27e-13
1	1.1E+08	rs646776	C	-2.55e-03	4.60e-04	3.11e-08
1	1.5E+08	rs1044808	C	-4.06e-03	7.17e-04	1.49e-08
1	1.5E+08	rs1694379	C	2.28e-03	3.96e-04	8.41e-09
1	1.51E+08	rs267738	G	-3.48e-03	4.73e-04	1.87e-13
1	1.54E+08	rs4129267	T	-2.71e-03	3.94e-04	5.63e-12
1	1.55E+08	rs12904	A	-1.94e-03	3.92e-04	7.48e-07
1	1.6E+08	rs3093059	G	5.39e-03	7.99e-04	1.57e-11
1	1.6E+08	rs7553007	A	-3.96e-03	4.10e-04	4.87e-22
1	1.62E+08	rs61804211	T	3.93e-03	6.36e-04	6.72e-10
1	2.02E+08	rs2250377	A	2.44e-03	4.11e-04	2.98e-09
1	2.3E+08	rs10489615	A	2.49e-03	3.96e-04	3.28e-10
2	622827	rs2867125	T	-2.99e-03	5.10e-04	4.72e-09
2	27730940	rs1260326	T	6.00e-03	3.97e-04	1.32e-51
2	28145159	rs17758075	T	-2.52e-03	4.35e-04	6.99e-09
2	28268742	rs115640879	T	5.25e-03	8.34e-04	3.11e-10
2	59294558	rs10172678	T	2.53e-03	3.95e-04	1.54e-10
2	1.03E+08	rs1997466	C	2.26e-03	3.86e-04	4.66e-09
2	1.03E+08	rs72995652	A	3.20e-03	5.53e-04	7.61e-09
2	1.21E+08	rs17050272	A	2.48e-03	3.95e-04	3.26e-10
2	1.66E+08	rs1128249	T	-2.44e-03	3.97e-04	7.27e-10
2	1.79E+08	rs17400325	C	-5.33e-03	9.99e-04	9.58e-08
2	2.03E+08	rs3731696	G	3.55e-03	5.92e-04	1.92e-09
2	2.12E+08	rs1047891	A	2.41e-03	4.15e-04	6.82e-09
2	2.19E+08	rs13005100	T	2.12e-03	3.94e-04	7.44e-08
2	2.27E+08	rs2943641	T	-3.46e-03	4.04e-04	9.31e-18
3	25357870	rs1483840	A	2.26e-03	3.88e-04	6.06e-09
3	38460062	rs6807940	C	-2.27e-03	4.02e-04	1.77e-08
3	49941436	rs2280406	A	2.09e-03	3.86e-04	6.05e-08
3	1.72E+08	rs572169	T	-2.98e-03	4.18e-04	9.91e-13
4	55526702	rs3819391	A	2.09e-03	3.88e-04	7.23e-08
4	77412140	rs13146355	A	3.31e-03	3.90e-04	2.33e-17
4	1E+08	rs1229984	T	-8.02e-03	1.16e-03	5.50e-12
4	1.04E+08	rs223404	C	-2.44e-03	3.85e-04	2.36e-10
4	1.47E+08	rs4835265	A	3.00e-03	5.28e-04	1.39e-08
5	39429049	rs10062979	T	-2.56e-03	4.23e-04	1.35e-09
5	43039793	rs1054428	C	2.44e-03	4.15e-04	4.05e-09
5	52193125	rs4074793	G	3.80e-03	7.36e-04	2.35e-07
5	55860866	rs3936510	T	2.74e-03	4.80e-04	1.11e-08
5	78440369	rs1717565	C	-2.16e-03	4.05e-04	9.73e-08
5	1.12E+08	rs4705762	C	2.24e-03	3.86e-04	6.27e-09
5	1.77E+08	rs6420094	G	3.49e-03	4.17e-04	5.90e-17
6	7205796	rs4960295	A	-2.96e-03	3.89e-04	2.74e-14
6	31318177	rs4394275	A	3.16e-03	4.63e-04	8.85e-12
6	32050544	rs3130287	C	-3.91e-03	5.50e-04	1.17e-12
6	37101853	rs9470491	A	2.80e-03	5.17e-04	5.70e-08
6	43758873	rs6905288	G	-2.29e-03	3.90e-04	4.26e-09
6	43785741	rs112222843	T	-6.39e-03	1.04e-03	7.12e-10
6	43788415	rs10046368	C	-2.28e-03	4.20e-04	5.66e-08
6	43806609	rs881858	G	-3.37e-03	4.17e-04	7.02e-16
6	98445008	rs4524616	C	-2.97e-03	3.89e-04	2.64e-14
6	1.27E+08	rs9388489	G	-3.09e-03	3.89e-04	2.05e-15
6	1.4E+08	rs668459	C	2.45e-03	3.91e-04	4.18e-10
6	1.5E+08	rs9371486	C	-2.19e-03	4.09e-04	8.49e-08
6	1.53E+08	rs9397587	A	2.26e-03	3.94e-04	1.03e-08
6	1.61E+08	rs316019	A	-3.92e-03	6.32e-04	5.42e-10
6	1.61E+08	rs3127573	G	3.43e-03	5.70e-04	1.87e-09
7	1270699	rs6950388	G	-3.48e-03	4.76e-04	2.69e-13
7	6779810	rs62439737	G	3.07e-03	5.25e-04	5.30e-09
7	23502974	rs12534093	A	1.88e-03	4.50e-04	3.08e-05

7	44231216	rs3757840	G	-2.50e-03	3.86e-04	9.69e-11
7	72982874	rs17145738	T	-4.79e-03	5.89e-04	4.29e-16
7	73020301	rs799157	T	5.27e-03	9.50e-04	2.96e-08
7	1.21E+08	rs17284988	C	-2.08e-03	3.89e-04	9.46e-08
7	1.28E+08	rs322812	C	-2.26e-03	3.88e-04	5.86e-09
7	1.3E+08	rs4731702	T	-2.15e-03	3.86e-04	2.53e-08
7	1.31E+08	rs157934	C	-2.93e-03	4.19e-04	2.66e-12
7	1.51E+08	rs7805747	A	3.35e-03	4.33e-04	9.19e-15
8	10771013	Affx-31289294	G	-2.52e-03	4.43e-04	1.34e-08
8	19727047	rs1441778	C	-2.96e-03	5.29e-04	2.22e-08
8	19777695	rs75218485	T	-8.39e-03	1.29e-03	7.35e-11
8	19805708	rs1801177	A	8.65e-03	1.46e-03	3.45e-09
8	19813529	rs268	G	7.88e-03	1.47e-03	8.29e-08
8	19855344	rs17489373	A	-5.16e-03	4.36e-04	2.85e-32
8	19941854	rs4543559	A	2.72e-03	3.98e-04	8.17e-12
8	23751151	rs10109414	T	2.15e-03	3.93e-04	4.69e-08
8	1.26E+08	rs6982502	C	3.88e-03	3.88e-04	1.58e-23
8	1.27E+08	rs2954038	C	4.65e-03	4.23e-04	3.82e-28
8	1.27E+08	rs7832357	G	2.42e-03	4.08e-04	3.11e-09
9	4840877	rs1270231	G	3.32e-03	6.09e-04	4.73e-08
9	1.03E+08	rs1226591	C	2.25e-03	3.92e-04	8.68e-09
9	1.17E+08	rs2636897	A	2.21e-03	3.87e-04	1.09e-08
10	17845995	rs117163080	C	3.55e-03	5.61e-04	2.38e-10
10	17891705	rs1926736	A	-2.69e-03	3.98e-04	1.37e-11
10	44539913	rs2047009	T	2.15e-03	3.87e-04	2.85e-08
10	71093392	rs16926246	T	-3.57e-03	5.75e-04	5.57e-10
10	1.02E+08	rs2862954	C	-3.19e-03	3.91e-04	3.26e-16
10	1.05E+08	rs284859	T	-2.96e-03	5.09e-04	6.31e-09
11	10259378	rs4910109	A	1.91e-03	3.87e-04	8.05e-07
11	15832567	rs1470144	T	2.37e-03	4.20e-04	1.61e-08
11	30346052	rs1222216	T	-2.52e-03	4.64e-04	5.74e-08
11	30754837	rs55733296	A	6.16e-03	1.02e-03	1.65e-09
11	30760335	rs3925584	C	-2.46e-03	3.89e-04	2.57e-10
11	32956492	rs62618693	T	-6.08e-03	9.47e-04	1.34e-10
11	33759092	rs11032362	A	-4.81e-03	6.80e-04	1.55e-12
11	43653833	rs2862996	G	2.63e-03	4.17e-04	2.71e-10
11	47306585	rs1051006	A	-4.17e-03	5.01e-04	9.31e-17
11	47440758	rs2293579	A	2.82e-03	3.96e-04	1.16e-12
11	55468388	rs78816858	T	3.43e-03	6.08e-04	1.60e-08
11	55563900	rs297055	C	-2.37e-03	4.36e-04	5.50e-08
11	64031241	rs35169799	T	4.63e-03	8.01e-04	7.82e-09
11	65458964	rs7115734	A	3.10e-03	4.07e-04	2.61e-14
11	72497462	rs481206	T	2.19e-03	4.06e-04	7.49e-08
11	1.17E+08	rs964184	G	8.10e-03	5.61e-04	3.06e-47
11	1.17E+08	rs7112513	A	4.27e-03	6.21e-04	6.52e-12
12	15344407	rs11056396	C	-3.57e-03	5.88e-04	1.32e-09
12	84152210	rs10862748	G	-2.17e-03	4.03e-04	7.51e-08
12	84974976	rs2896498	C	2.47e-03	4.63e-04	9.20e-08
12	1.03E+08	rs11111274	G	-3.00e-03	4.32e-04	3.71e-12
12	1.12E+08	rs3184504	T	2.71e-03	3.91e-04	4.05e-12
12	1.21E+08	rs7310409	A	-5.11e-03	3.97e-04	7.18e-38
12	1.21E+08	rs117968973	T	5.53e-03	1.01e-03	4.84e-08
14	50655357	rs72681869	C	-1.63e-02	1.90e-03	1.08e-17
14	69599483	rs12432645	T	2.48e-03	4.44e-04	2.35e-08
14	94286525	rs12588988	C	-2.11e-03	3.86e-04	4.55e-08
15	41410705	rs1678750	A	2.32e-03	3.87e-04	1.97e-09
15	42032383	rs17677991	G	2.38e-03	4.06e-04	5.02e-09
15	43820717	rs55707100	T	7.65e-03	1.24e-03	6.23e-10
15	45654327	rs1145086	G	4.07e-03	4.01e-04	3.32e-24
15	51524292	rs2414095	A	1.88e-03	4.04e-04	3.45e-06
15	60878030	rs340005	G	-2.66e-03	3.97e-04	1.94e-11
15	63396301	rs2652836	C	2.59e-03	4.56e-04	1.35e-08
15	72241857	rs1481860	T	-2.40e-03	4.48e-04	8.51e-08
15	74328116	Affx-11961369	G	2.63e-03	3.85e-04	8.00e-12
15	76508632	rs80292319	C	-4.52e-03	8.41e-04	7.82e-08
16	1129010	rs4988483	A	6.87e-03	8.83e-04	7.53e-15
16	3586230	rs9790	T	2.64e-03	4.85e-04	5.51e-08
16	20364588	rs4293393	G	-3.41e-03	4.96e-04	6.50e-12
16	31056433	rs35468353	G	2.20e-03	3.98e-04	3.47e-08
16	51172677	rs11645288	A	3.12e-03	4.89e-04	1.80e-10
16	53800754	rs9940128	A	2.38e-03	3.90e-04	9.66e-10

16	56990716	rs247617	A	-4.89e-03	4.13e-04	2.21e-32
16	57006590	rs7499892	T	5.34e-03	5.00e-04	9.78e-27
16	69575238	rs62052820	A	3.70e-03	4.64e-04	1.39e-15
16	69588572	rs1364063	C	-2.74e-03	3.94e-04	3.72e-12
16	72114002	rs217181	T	-2.69e-03	4.88e-04	3.43e-08
16	79749353	rs3813582	C	2.36e-03	4.16e-04	1.35e-08
16	81534790	rs2925979	T	3.04e-03	4.20e-04	4.28e-13
16	89818491	rs3743860	C	-2.21e-03	3.91e-04	1.70e-08
17	19440538	rs894680	A	2.21e-03	4.00e-04	3.06e-08
17	19474875	rs111653425	T	1.23e-02	1.82e-03	1.23e-11
17	34876195	rs11657469	T	-2.34e-03	3.88e-04	1.79e-09
17	37626963	rs11657899	G	-2.57e-03	4.47e-04	9.06e-09
17	40722029	rs665268	G	2.59e-03	4.29e-04	1.47e-09
17	41470683	rs4793248	A	-2.41e-03	3.98e-04	1.37e-09
17	47109998	rs9916472	T	-2.19e-03	4.11e-04	9.98e-08
17	59456589	rs9895661	C	3.32e-03	5.04e-04	4.57e-11
17	76796463	rs11077397	C	2.07e-03	3.88e-04	9.69e-08
18	21117419	rs4800162	T	-2.12e-03	3.86e-04	3.75e-08
18	56095378	rs4940691	T	3.20e-03	4.78e-04	2.05e-11
18	56152053	rs12606449	C	-2.53e-03	4.36e-04	6.18e-09
18	57735945	rs36030660	C	2.37e-03	4.34e-04	4.61e-08
19	8429323	rs116843064	A	-9.20e-03	1.41e-03	7.83e-11
19	33364628	rs8101881	C	-2.25e-03	3.93e-04	1.06e-08
19	33899065	rs731839	G	2.76e-03	4.07e-04	1.32e-11
19	45410002	rs769449	A	-4.60e-03	5.92e-04	7.28e-15
19	49206462	rs681343	C	-3.71e-03	3.88e-04	9.95e-22
20	25210827	rs6115094	G	2.12e-03	3.86e-04	4.25e-08
20	26176538	rs117281921	T	-5.31e-03	8.77e-04	1.45e-09
20	39142516	rs2207132	A	5.98e-03	1.09e-03	3.79e-08
20	62470872	rs4809370	T	-2.43e-03	3.94e-04	6.88e-10
20	62712053	rs8121509	C	-3.01e-03	3.88e-04	8.27e-15
20	62761699	rs6062643	G	2.29e-03	3.82e-04	1.92e-09
22	24995668	rs2006092	G	3.17e-03	4.07e-04	6.92e-15
22	29088123	rs5762746	T	-2.39e-03	3.99e-04	2.32e-09
22	38569006	rs738322	G	-2.07e-03	3.86e-04	8.66e-08
22	44324727	rs738409	G	4.69e-03	4.67e-04	8.25e-24

Supplementary Table 15 Results from the two-step least squares Mendelian randomisation approach using instrumental variables detected using the BOLT-LMM model (A, N=158 instruments explaining 1.9% of the BHS), a more stringent p-value threshold of 10^{-8} (B, N=163 instruments explaining 2.0% of the BHS), a more stringent r^2 of 0.01 (C, N=134 explaining 1.8% of the BHS). Causal effects (β) were estimated using a proportional hazard Cox model from a regression of the instrumentally explained BHS against all-cause, cancer and CVD mortality, and cancer and CVD incidence. Hazard Ratios (HRs) are expressed for a 0.1 increase in the score, and we report the p-value assessing if the causal effect is different from 0. We present estimates for the model adjusted for age, sex and the first 10 principal components capturing the latent structure of the UK Biobank population (Base model), and for the model additionally adjusted for education. Results from the multivariable Mendelian randomisation were adjusted for age, sex and the 10 first principal components capturing the latent structure of the UK Biobank population.

A

	Base model			Base model + Education			Base model + $\widehat{\text{Education}}$		
	β	HR	p-value	β	HR	p-value	β	HR	p-value
All-cause mortality	0.04	1.04	5.14×10^{-1}	0.02	1.02	7.32×10^{-1}	0.03	1.03	6.04×10^{-1}
Cancer mortality	0.02	1.02	8.35×10^{-1}	-0.01	0.99	8.74×10^{-1}	0.01	1.01	8.96×10^{-1}
CVD mortality	0.15	1.16	3.48×10^{-1}	0.13	1.14	4.08×10^{-1}	0.14	1.15	3.74×10^{-1}
Cancer incidence	0.01	1.01	5.01×10^{-1}	0.01	1.01	6.12×10^{-1}	0.01	1.01	5.31×10^{-1}
CVD incidence	0.26	1.29	1.07×10^{-9}	0.24	1.28	7.43×10^{-9}	0.25	1.28	2.80×10^{-9}

B

	Base model			Base model + Education			Base model + $\widehat{\text{Education}}$		
	β	HR	p-value	β	HR	p-value	β	HR	p-value
All-cause mortality	0.04	1.04	4.38×10^{-1}	0.02	1.02	7.35×10^{-1}	0.03	1.03	5.42×10^{-1}
Cancer mortality	0.00	1.00	1.00×10^{0}	-0.03	0.97	6.49×10^{-1}	-0.01	0.99	9.24×10^{-1}
CVD mortality	0.15	1.16	3.25×10^{-1}	0.13	1.14	3.87×10^{-1}	0.14	1.15	3.57×10^{-1}
Cancer incidence	0.01	1.01	5.40×10^{-1}	0.01	1.01	6.39×10^{-1}	0.01	1.01	5.77×10^{-1}
CVD incidence	0.26	1.30	2.11×10^{-10}	0.25	1.29	1.95×10^{-9}	0.25	1.29	7.40×10^{-10}

C

	Base model			Base model + Education			Base model + $\widehat{\text{Education}}$		
	β	HR	p-value	β	HR	p-value	β	HR	p-value
All-cause mortality	0.05	1.06	3.27×10^{-1}	0.03	1.03	5.53×10^{-1}	0.04	1.05	4.18×10^{-1}
Cancer mortality	-0.01	0.99	8.95×10^{-1}	-0.04	0.96	6.26×10^{-1}	-0.02	0.98	8.18×10^{-1}
CVD mortality	0.23	1.26	1.45×10^{-1}	0.22	1.24	1.80×10^{-1}	0.22	1.25	1.64×10^{-1}
Cancer incidence	0.02	1.02	4.96×10^{-1}	0.01	1.01	6.36×10^{-1}	0.01	1.01	5.32×10^{-1}
CVD incidence	0.21	1.23	2.15×10^{-6}	0.19	1.21	1.17×10^{-5}	0.20	1.22	5.67×10^{-6}

Supplementary Figure 1: Flowchart of participants selection

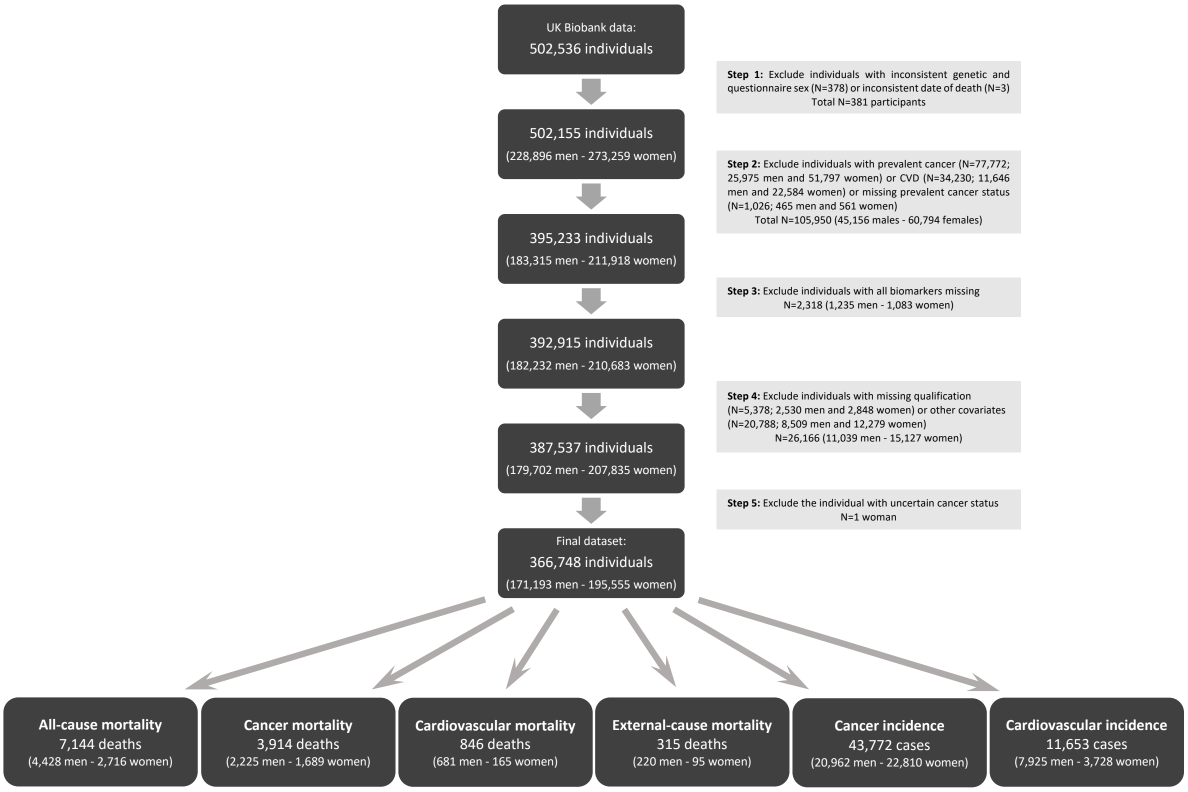

Supplementary Figure 2 Regression coefficients from the proportional hazard Cox model relating all-cause, cancer, and cardiovascular mortality, cancer and cardiovascular disease incidence, and (i) the standardised levels of (i) the BHS and the score first principal component from the 13 biomarkers (red), (ii) the 4 biomarkers from the metabolic system (blue), (iii) the 3 biomarkers from the cardiovascular system (green), (iv) the 2 biomarkers from the inflammation system (purple), (v) the 3 biomarkers from the liver system (orange), and (vi) creatinine, the only biomarker in the kidney system (black). Results are presented for men (left) and women (right) and for the model adjusted for education group, behaviours and lifestyle (smoking, physical activity, and alcohol consumption), BMI, and comorbidities and treatments.

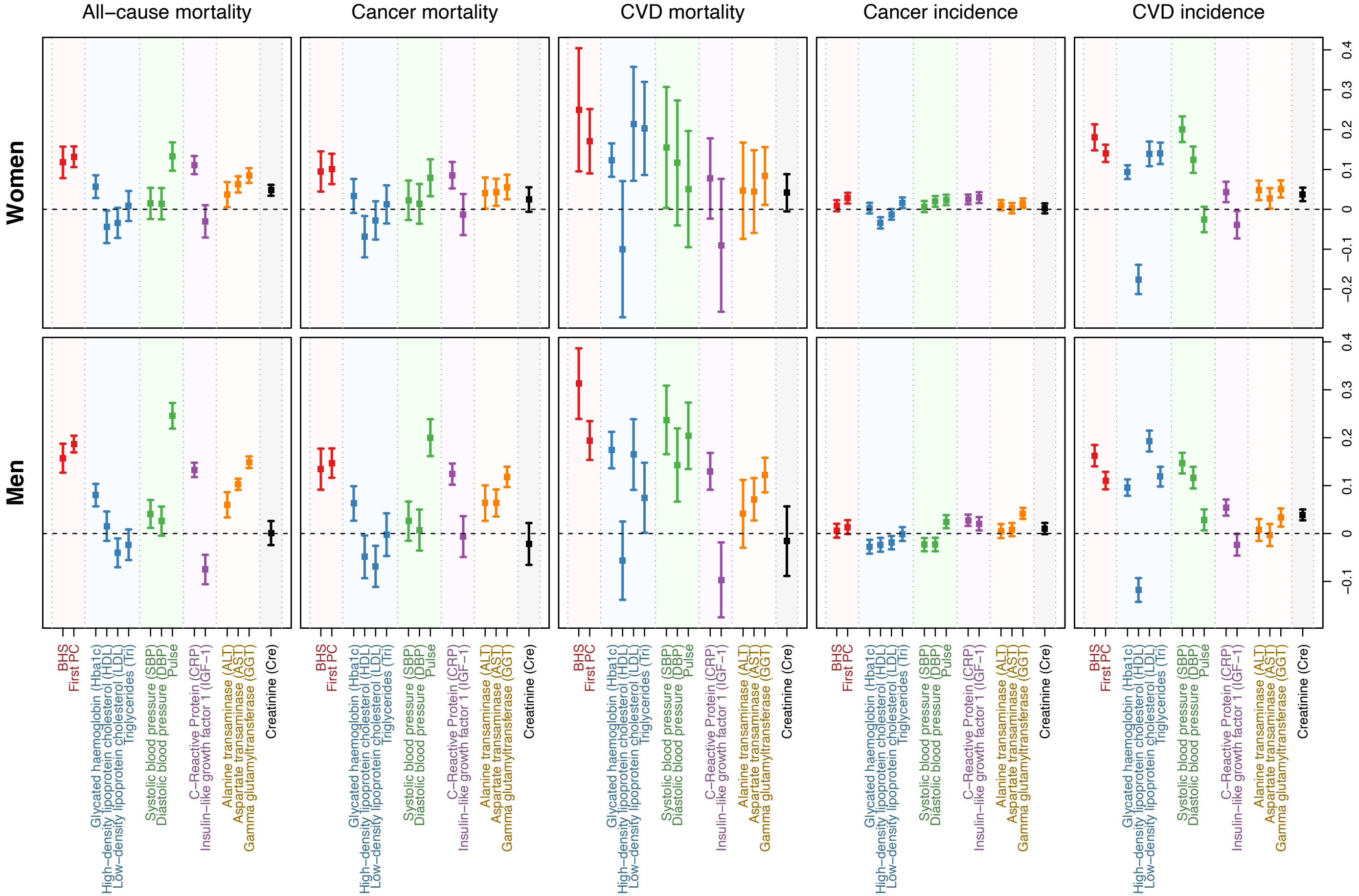

Supplementary Figure 3 Hazard ratio from the proportional hazard Cox model relating external-cause mortality and the BHS (red), the metabolic (blue), the cardiovascular (green), the inflammatory (purple), the kidney (orange), and the liver (grey) sub-scores. Hazard ratios are expressed as a risk change per 0.1 increase in the score. Results are presented for men (left) and women (right) and for the unadjusted model, and for models sequentially adjusted for education, behaviours and lifestyle (smoking, physical activity, and alcohol consumption), BMI, and numbers of comorbidities and treatments.

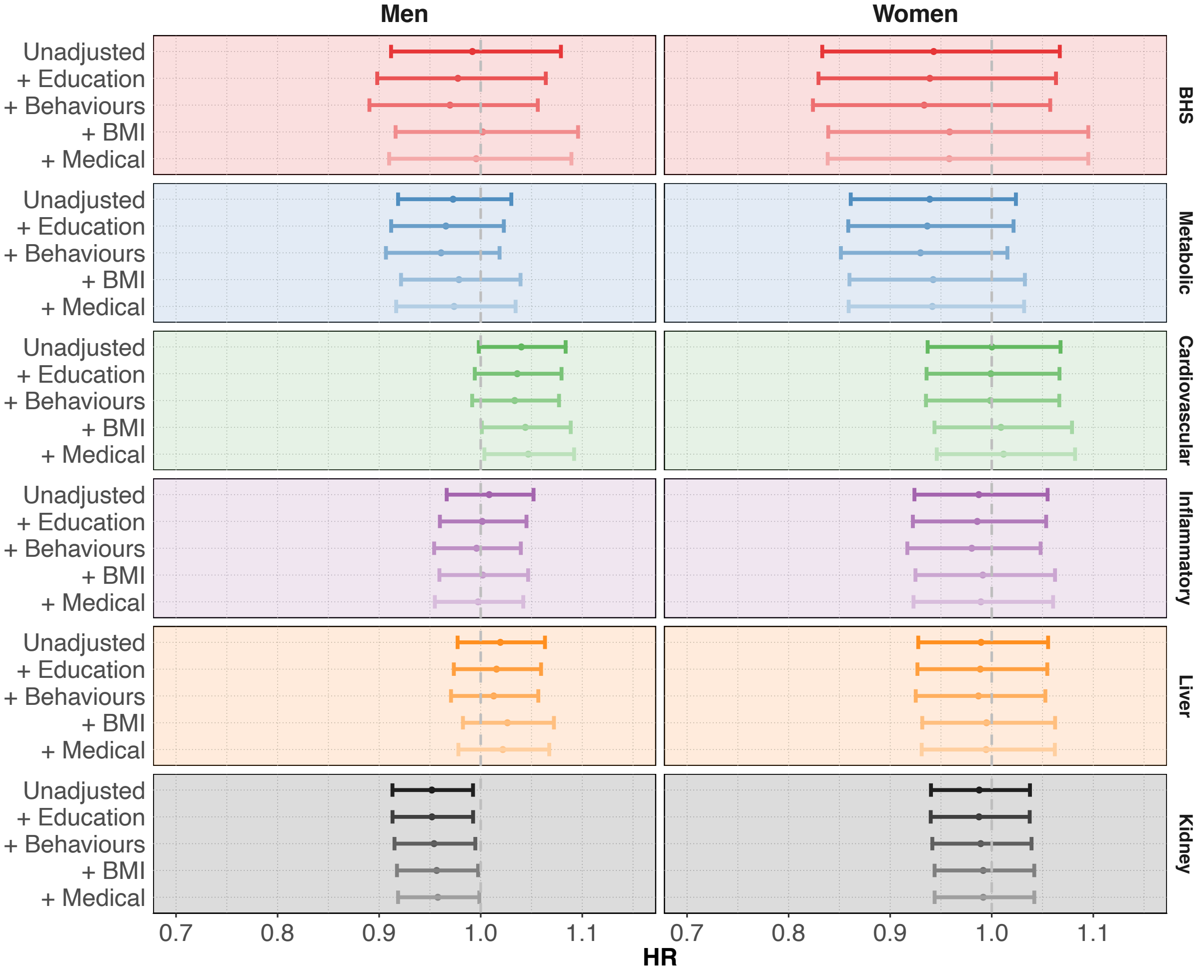

Supplementary Figure 4: Hazard ratio from the proportional hazard Cox model relating (A) all cause, (B) cancer and (C) cardiovascular mortality, cancer (D), and CVD (E) incidence and the Biological Health Score (BHS, red), the metabolic (blue), the cardiovascular (green), the inflammatory (purple), the kidney (orange), and the liver (grey) sub-scores. Hazard ratios are expressed as a risk change per 0.1 increase in the score. Results are presented for men (left) and women (right) and for the model adjusted for ethnicity (coded as White, Black, and Other ethnicity), for models sequentially adjusted for education group, lifestyle behaviours (smoking, physical activity, and alcohol consumption), BMI and medical status (number of comorbidities and treatments)

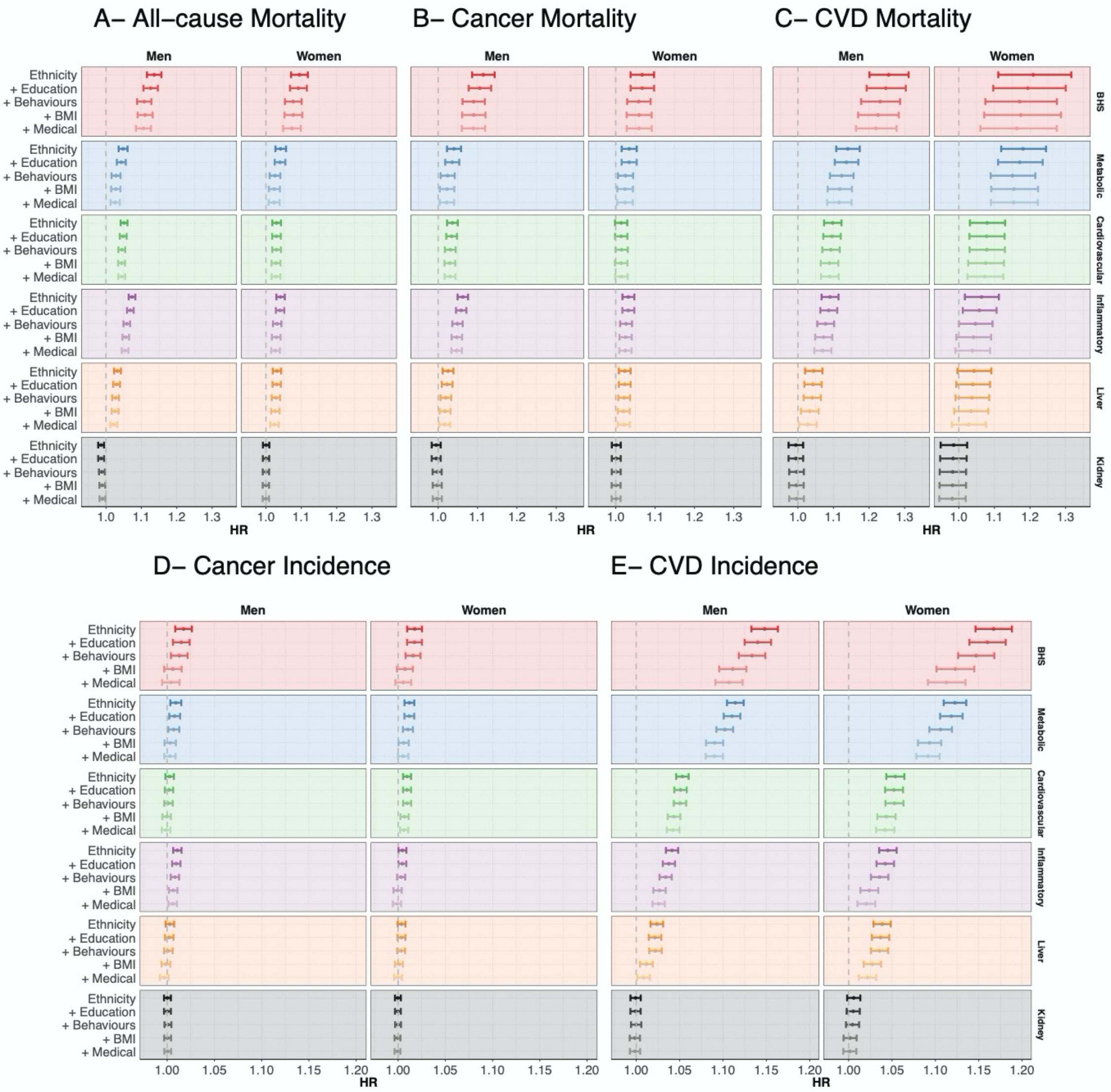

Supplementary Figure 5 Hazard ratio from the proportional hazard Cox model relating all-cause (A), cancer (B), and cardiovascular (C) mortality and, as a continuous alternative to (i) the BHS, the scores of the first component of 13 biomarkers (red), (ii) the metabolic score, the scores of the first component from the 4 included biomarkers (blue), (iii) the cardiovascular score, the scores of the first component from the 3 included biomarkers (green), (iv) the inflammatory score, the scores of the first component from the 2 inflammation-related biomarkers (purple), (v) the kidney score, the standardised levels of creatinine (the only biomarker in that system) (orange), and (vi) the liver score, the scores of the first component from the 3 included biomarkers (grey). Hazard ratios are expressed as a risk change per 0.1 increase in the score. Results are presented for men (left) and women (right) and for the unadjusted model, and for models sequentially adjusted for education group, behaviours and lifestyle (smoking, physical activity, and alcohol consumption), BMI, and numbers of comorbidities and treatments.

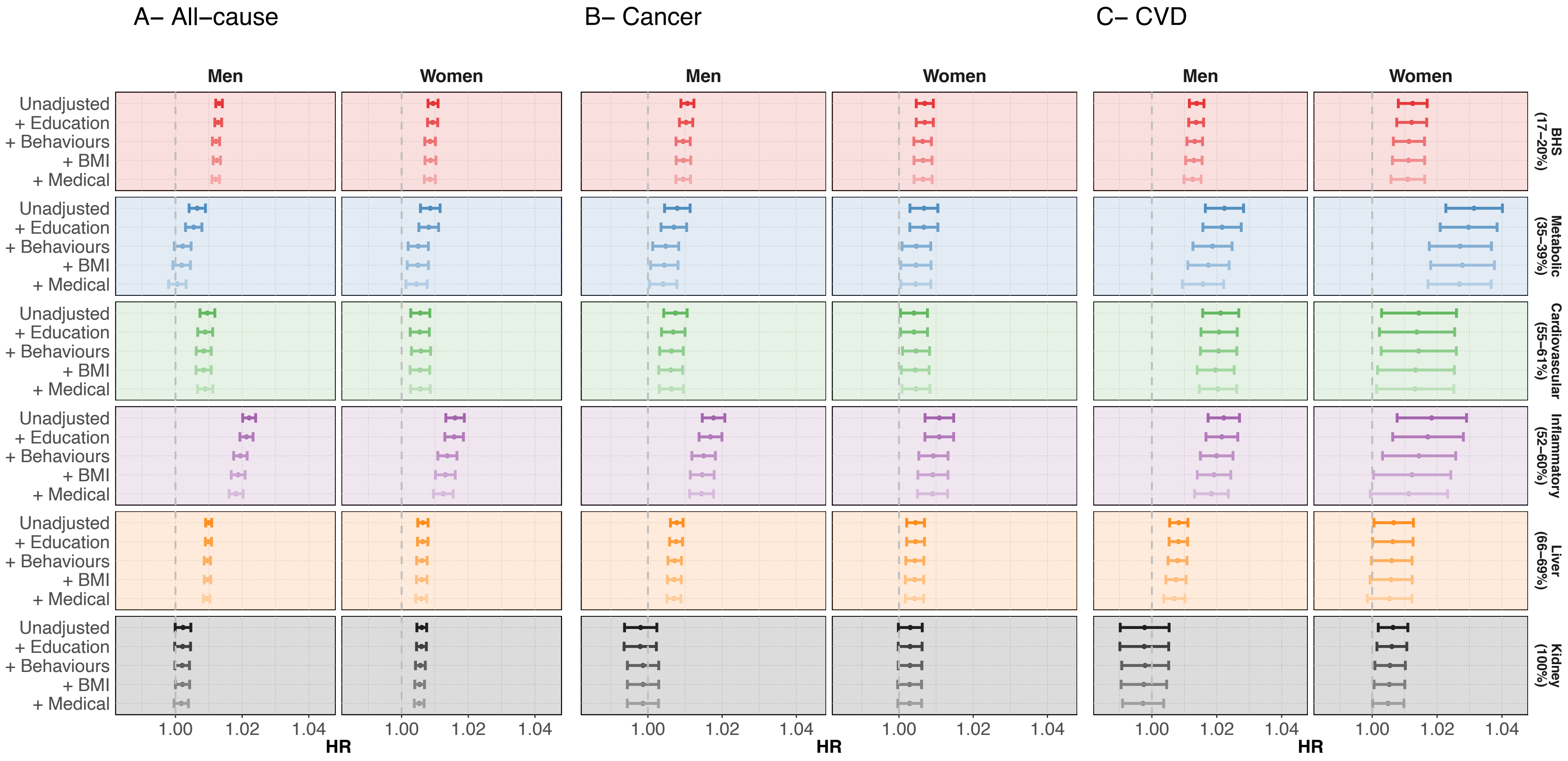

Supplementary Figure 6 Hazard ratio from the proportional hazard Cox model relating cancer (A), and cardiovascular (B) incidence and, as a continuous alternative to (i) the BHS, the scores of the first component of 13 biomarkers (red), (ii) the metabolic score, the scores of the first component from the 4 included biomarkers (blue), (iii) the cardiovascular score, the scores of the first component from the 3 included biomarkers (green), (iv) the inflammatory score, the scores of the first component from the 2 inflammation-related biomarkers (purple), (v) the kidney score, the standardised levels of creatinine (the only biomarker in that system) (orange), and (vi) the liver score, the scores of the first component from the 3 included biomarkers (grey). Hazard ratios are expressed as a risk change per 0.1 increase in the score. Results are presented for men (left) and women (right) and for the unadjusted model, and for models sequentially adjusted for education group, behaviours and lifestyle (smoking, physical activity, and alcohol consumption), BMI, and numbers of comorbidities and treatments.

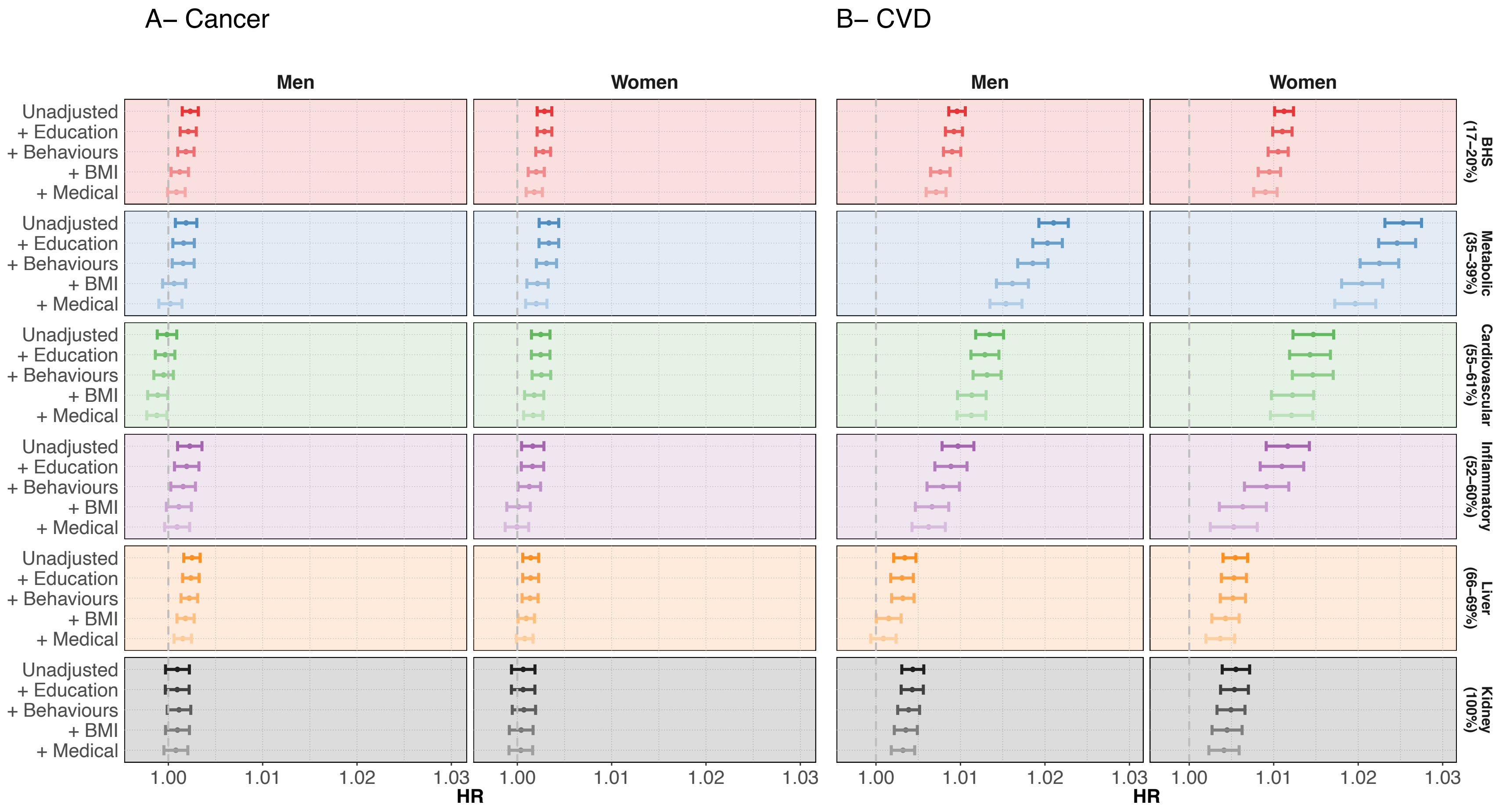

Supplementary Figure 7 Schoenfeld residuals plots of the BHS from Cox proportional hazards models relating all-cause, cancer, CVD and external-causes mortality to the BHS in the unadjusted and fully adjusted models. Results are presented in men (A) and women (B), separately. Splines with 4 degrees of freedom are fitted on the residuals (plain line) and surrounded by the 2-standard-error band (delimited by the dashed lines).

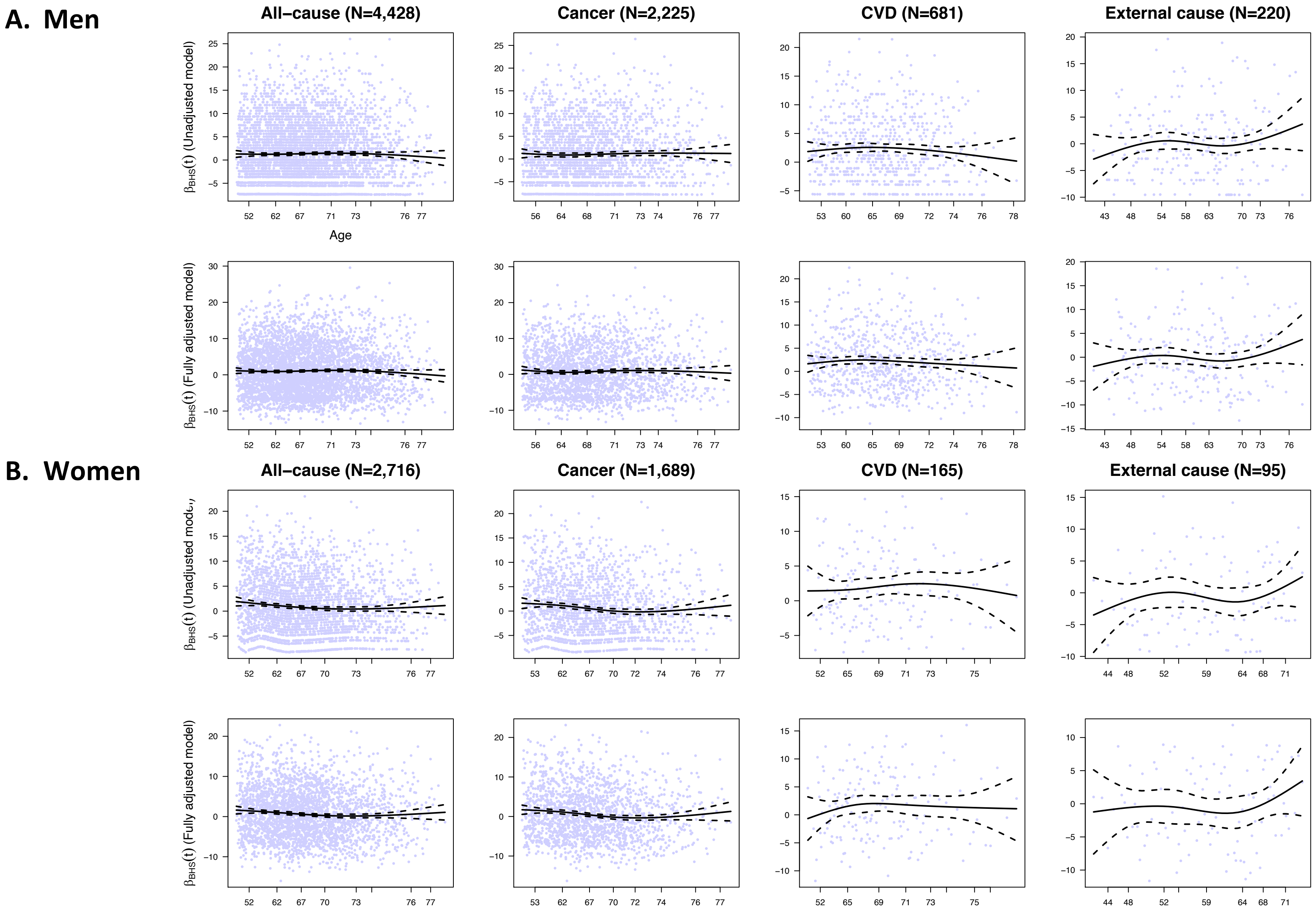

Supplementary Figure 8 Schoenfeld residuals plots of the BHS from Cox proportional hazards models relating cancer, and CVD incidence to the BHS in the unadjusted and fully adjusted models. Results are presented in men (A) and women (B), separately. Splines with 4 degrees of freedom are fitted on the residuals (plain line) and surrounded by the 2-standard-error band (delimited by the dashed lines).

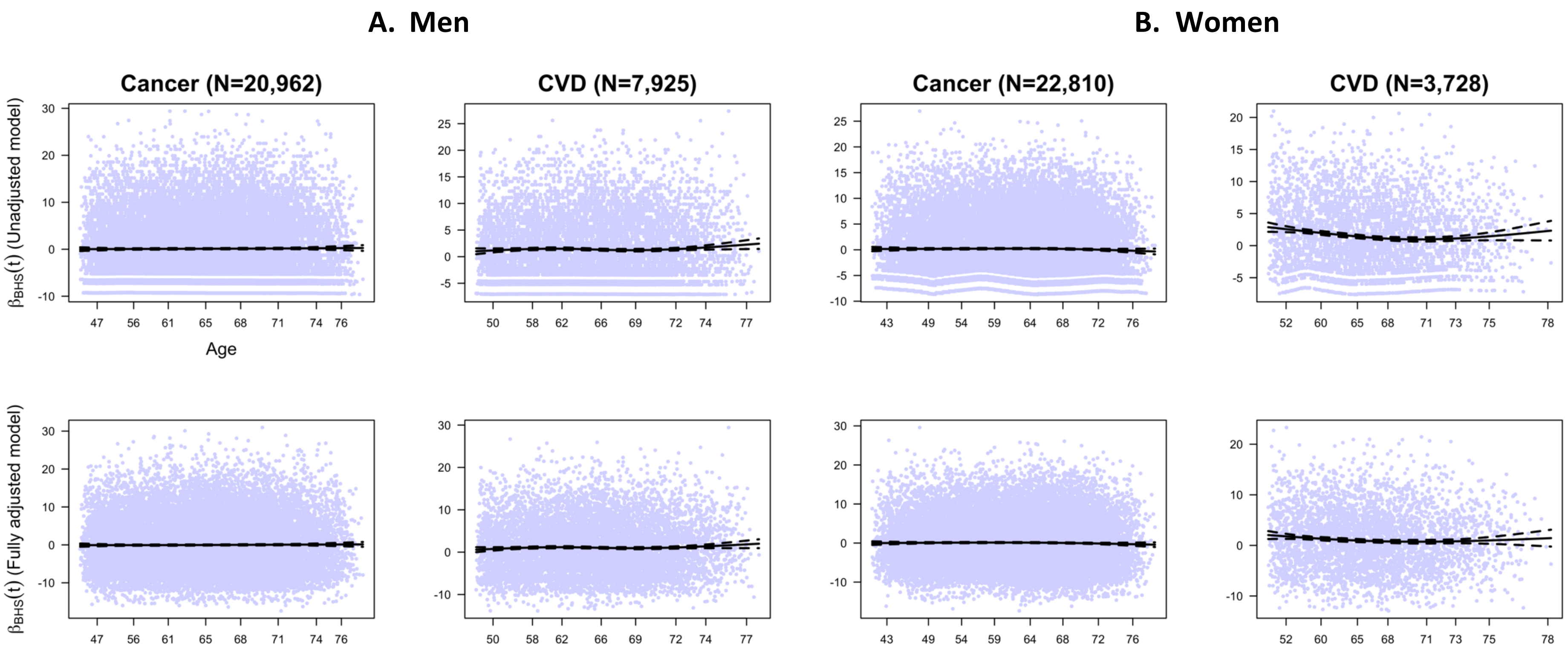

Supplement: Supplementary file 1 [file mmc1.pdf]
